# NR2F6 functions as a key transcriptional checkpoint enforcing dysfunction of human CD3^+^ T cells and limiting CAR-T cell potency

**DOI:** 10.1016/j.isci.2026.117982

**Published:** 2026-10-26

**Authors:** Jiří Koutník, Thomas Gruber, Dorothee Freimark, Dominik Humer, Daniel Schreiber, Isabel Hölzl, Viktor Lang, Nino Tokic, Tajana Sajinovic, Dominik Wolf, Nikolaus Thuille, Kerstin Siegmund, Victoria Klepsch, Gottfried Baier

**Affiliations:** 1 Institute of Cell Genetics, Medical University of Innsbruck, 6020 Innsbruck, Austria; 2 Internal Medicine V (Hematology and Oncology), Comprehensive Cancer Center Innsbruck (CCCI), Tyrolean Cancer Research Institute (TKFI), Medical University of Innsbruck, 6020 Innsbruck, Austria

**Keywords:** NR2F6, T cell dysfunction, immune checkpoint, transcriptional regulation, TCF-1/TCF7, CAR-T cells

## Abstract

Although nuclear receptor subfamily 2 group F member 6 (NR2F6) regulates lymphocyte activation in murine models, its function in human T cells remains incompletely defined. Here, we identify NR2F6 as a T cell receptor (TCR)-inducible transcriptional checkpoint that limits effector potency in human CD3^+^ T cells, including chimeric antigen receptor (CAR) T cells. In an anti-EGFR CAR-T cell-A549 co-culture model, enforced *NR2F6* expression, mimicking its upregulation in tumor-infiltrating lymphocytes, markedly impairs killing under repeated tumor challenge. In polyclonal T cells, *NR2F6* overexpression and CRISPR-Cas9-mediated ablation produced directionally opposing effects, supporting its role as a negative regulator of activation, differentiation-associated programs, and cytokine production. Mechanistically, transcriptomic, epigenomic, and nuclear protein analyses are consistent with suppression of NFAT-, AP-1-, and NF-κB-associated pathways and reveal context-dependent changes in TCF-1-associated readouts. Collectively, these findings position NR2F6 as a signaling-integrated transcriptional checkpoint that contributes to dysfunctional T cell states and may promote tumor immune evasion in humans.

## Highlights

• NR2F6’s suppressive function is conserved in human T cells
• Broad T cell inhibition by NR2F6 affects NFAT, AP-1, and NF-κB signaling
• NR2F6 context-dependently modulates TCF-1-associated programs
• Elevated NR2F6 limits CAR-T cell killing during serial tumor challenge

## Graphical abstract

**Figure:**
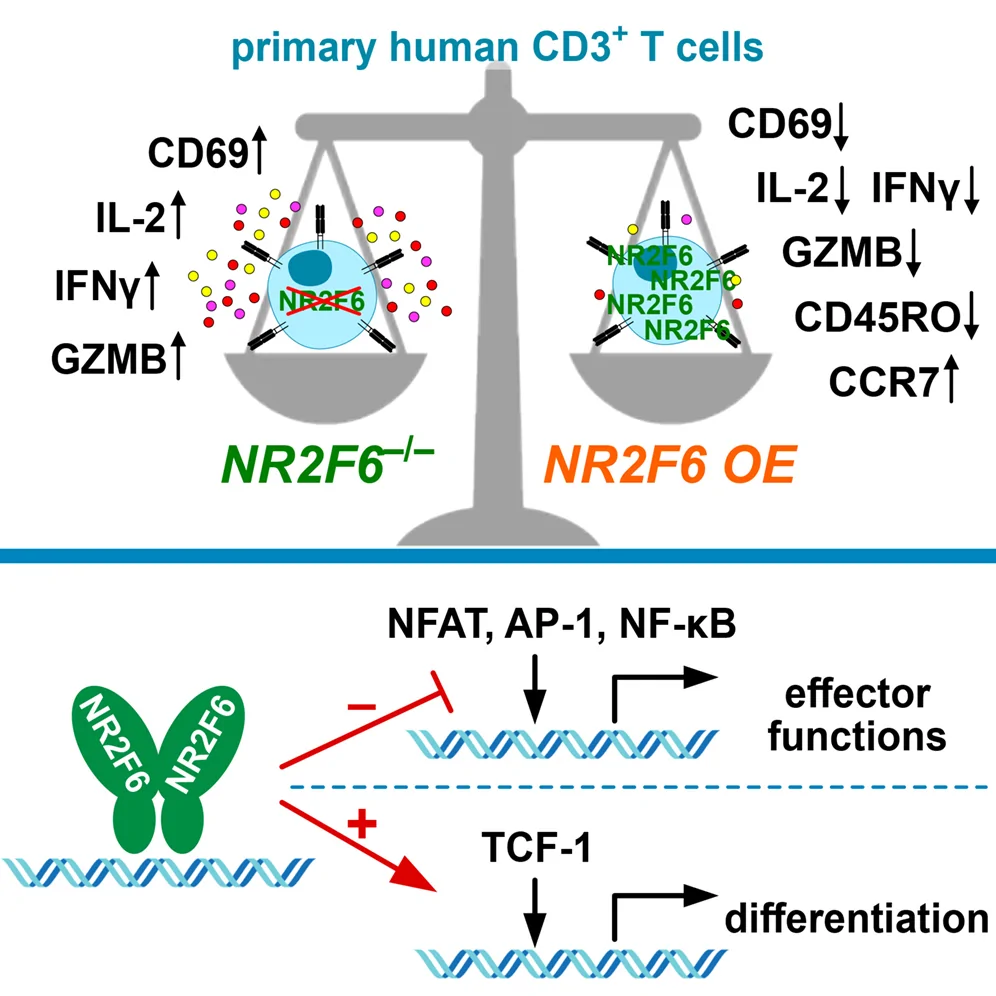

## Teaser

Immunology; Cell biology

## Introduction

The identification of novel targets that restrain anti-tumor immunity represents a critical challenge in cancer immunotherapy. Despite transformative advances achieved with immune checkpoint blockade and adoptive cell therapies, a substantial proportion of patients fail to mount durable responses, highlighting the need to overcome immune evasive mechanisms and to more effectively boost the cancer immunity cycle.^1^^,^^2^^,^^3^ Among the dominant barriers to sustained anti-tumor responses is T cell dysfunction, a progressive state characterized by impaired effector function, altered differentiation trajectories, and the establishment of transcriptional programs that limit cytotoxic capacity within the tumor microenvironment.

Recent evidence from murine models has established the orphan nuclear receptor NR2F6 (nuclear receptor subfamily 2 group F member 6) as a potent negative regulator of T cell responses and a key tumor immunity checkpoint. Genetic *Nr2f6* ablation in mice dramatically enhances anti-tumor immunity and synergizes with immune checkpoint blockade, underscoring its causal role in cancer immunity.^4^^,^^5^^,^^6^^,^^7^^,^^8^^,^^9^ Notably, *NR2F6* upregulation in whole-tumor tissue is correlated with poor prognosis across several human malignancies,^10^^,^^11^^,^^12^^,^^13^^,^^14^^,^^15^^,^^16^ and high expression in tumor-infiltrating lymphocytes (TILs) has been demonstrated in non-small cell lung cancer (NSCLC).^6^ The latter finding suggests that tumor-intrinsic mechanisms drive *NR2F6* expression as an immune-evasive strategy.

Mechanistically, NR2F6 has been proposed to function as a transcriptional repressor that dampens T cell receptor (TCR)-induced cytokine production and effector gene expression. In murine T cells, NR2F6 interferes with the accessibility and activity of key transcriptional regulators downstream of TCR signaling, thereby setting a threshold for activation.^4^^,^^5^^,^^17^ However, T cell responses are not solely defined by acute activation events. They are governed by tightly coordinated fate decisions that determine whether cells adopt effector, memory, progenitor exhausted, or terminally dysfunctional states. These lineage trajectories are orchestrated by interconnected transcription factor networks and are profoundly shaped by persistent antigen stimulation in cancer.

Whether NR2F6 contributes to the establishment or stabilization of dysfunctional T cell states and how it interfaces with core fate determinants, remains incompletely understood, particularly in the human system. Addressing these questions is essential for evaluating the translational potential of NR2F6 as a therapeutic target, especially in the context of engineered T cell therapies where intrinsic checkpoints can limit efficacy (as described by Chen et al.,^18^ Freitas et al.,^19^ or Kumar et al. and Wang et al.^20^^,^^21^).

To address this gap, we employed a lentiviral genetic perturbation strategy coupled with antibiotic selection to enable robust modulation of *NR2F6* in primary human T cells. Using this platform, we systematically interrogated NR2F6 function across orthogonal readouts, including chimeric antigen receptor (CAR)-T cell assays, flow cytometry, RNA sequencing, and transcription factor activity analyses. Our findings reveal that NR2F6-mediated suppression of T cell function is evolutionarily conserved between mice and humans and that NR2F6 acts as a TCR-inducible, reinforcing transcriptional checkpoint that broadly restrains activation, effector programs, and differentiation in human T cells. Moreover, we identify context-dependent changes in T cell factor 1 (TCF-1)-associated readouts following NR2F6 perturbation, without establishing direct molecular regulation. Collectively, these results establish NR2F6 as a central checkpoint governing human T cell immunity and position it as a compelling therapeutic target in immuno-oncology.

## Results

### NR2F6 limits CAR-T cell functions in an *in vitro* NSCLC model

Based on previous studies identifying murine NR2F6 as a transcriptional repressor of T cell effector function^4^^,^^5^ and its strong induction within the human lung cancer microenvironment,^6^ we hypothesized that NR2F6 represents a critical intrinsic limitation to CAR-T cell efficacy in solid tumors. To model the tumor-associated induction of NR2F6, we enforced *NR2F6* expression in CAR-T cells, which resulted in a profound impairment of CAR-T cell potency (Figure 1). Guided by prior reports implicating NR2F6 in NSCLC and the associated unmet clinical need, we employed an anti-epidermal growth factor receptor (EGFR) CAR targeting A549 tumor cells. Successful generation of CAR-T cells and *NR2F6* overexpression (OE) was confirmed by flow cytometry or immunoblot, respectively (Figures 1A–1C). Notably, during CAR-T cell production, *NR2F6* OE markedly reduced levels of activation markers (CD69 and CD25) while concomitantly altering the expression of activation-associated inhibitory receptors, including programmed cell death protein 1 (PD-1), lymphocyte activation gene 3 (LAG-3), and T-cell immunoglobulin and mucin-domain containing-3 (TIM-3), indicating a strongly attenuated activation state (Figure 1D). Upon antigen-specific restimulation with recombinant EGFR, *NR2F6* OE CAR-T cells exhibited an almost complete abrogation of effector cytokine production (Figure 1E). Consistently, functional impairment was further substantiated in a serial *in vitro* killing assay, where defective tumor cell lysis became increasingly pronounced with successive rounds of restimulation with fresh tumor targets, paralleled by an accumulating cytokine deficit in the co-culture supernatant (Figures 1F and 1G).

**Figure 1:**
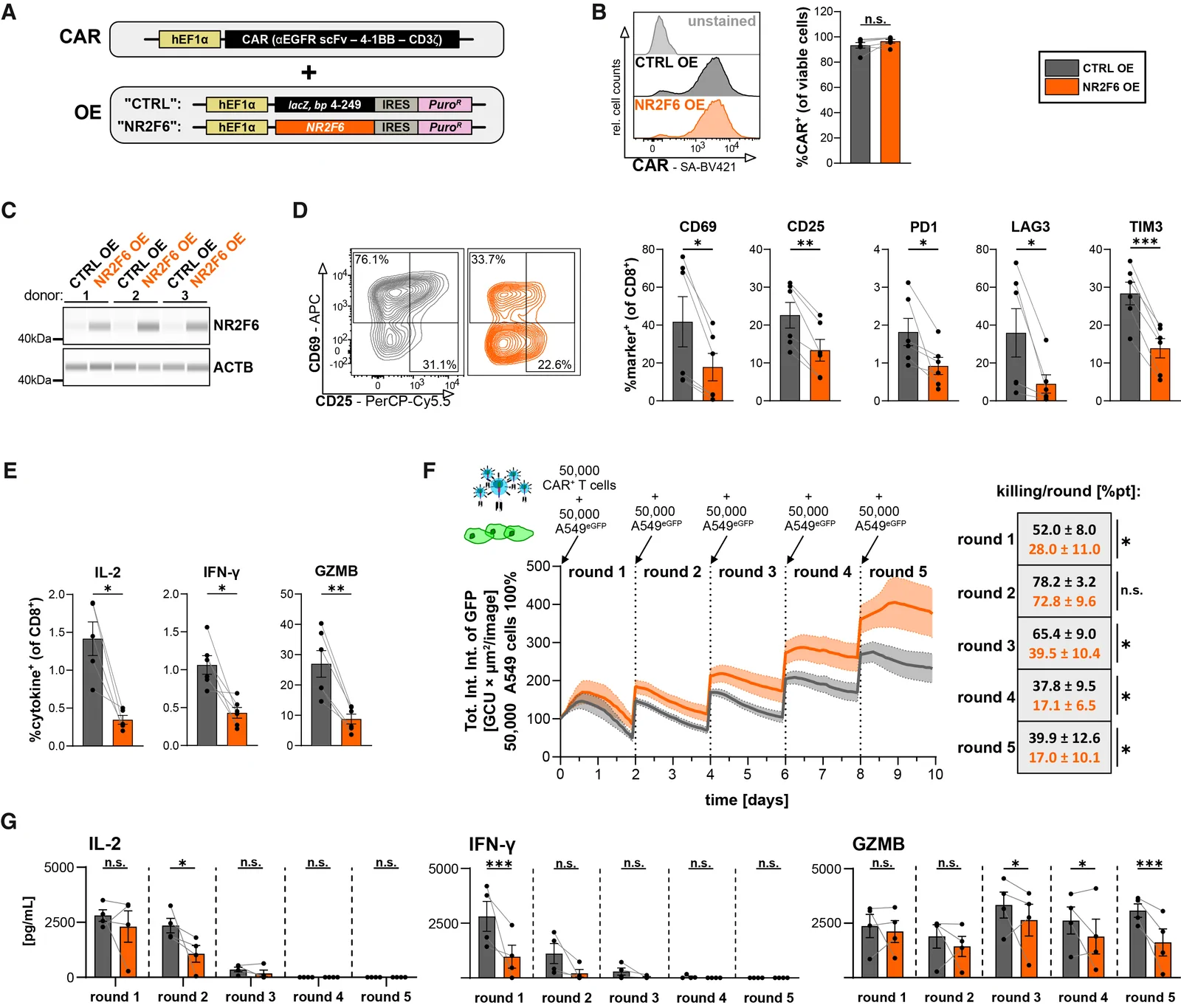
NR2F6 limits CAR-T cell functions in an *in vitro* NSCLC model (A) Schematic overview of constructs used for CAR as well as *NR2F6* overexpression (orange) or the corresponding control (gray). (B) Flow cytometric confirmation of CAR expression. (*n* = 6 donors, paired *t* test). (C) Immunoblot for confirmation of *NR2F6* overexpression. (representative of *n* = 3 donors). (D) Flow cytometric characterization of activation markers (CD69 and CD25) and activation-related inhibitory receptors (PD-1, LAG-3, and TIM-3) at the end of CAR-T cell production. (*n* = 6 donors, paired *t* test). (E) Intracellular flow cytometry for analysis of IL-2, IFN-γ, and GZMB production upon 4 h of re-stimulation via 1 μg/mL plate-coated rEGFR in the presence of brefeldin A and monensin (added 45 min post re-stimulation). (*n* = 6 donors, paired *t* test). (F) Serial killing assay: CAR-T cells were co-cultured with A549^*B2M*−/− eGFP^ target cells at an effector-to-target (E:T) ratio of 1:1. Every 48 h, fresh A549^*B2M*−/− eGFP^ cells were added at the same number as initially seeded. Tumor cell killing was monitored by live-cell imaging every 2 h. (G) Bioplex analysis measuring IL-2, IFN-γ, and GZMB concentrations from supernatant of co-culture at the end of every round of killing, prior to re-stimulation with fresh tumor cells. (*n* = 7 donors). Killing of each round and bioplex were analyzed using repeated-measures two-way ANOVA, followed by a post hoc test with Holm-Šídák correction for multiple comparisons. All data are represented as mean ± SEM together with connected individual donor data points. n.s., not significant; ∗*p* < 0.05, ∗∗*p* < 0.01, ∗∗∗*p* < 0.001.

To rigorously define NR2F6’s functional role, we also explored the impact of *NR2F6* deficiency in CAR-T cells. However, a functional benefit was not anticipated in this setting, as NR2F6 is only weakly induced in control CAR-T cells under standard *in vitro* conditions, and sustained loss of an intrinsic inhibitory regulator is known to impose technical limitations due to restimulation-induced cell death, as described for other immune checkpoints such as PD-1.^22^^,^^23^^,^^24^ Using established model systems, we used a CRISPR-Cas9-mediated *NR2F6* knockout (KO) strategy that allowed for the enrichment of modified cells. For this purpose, sgRNAs were designed and first validated in an *in vitro* cleavage assay and in strongly *NR2F6*-expressing HEK293T cells, followed by transfer of the system into a lentiviral vector conferring puromycin resistance. Using this approach in Jurkat cells, we confirmed that *NR2F6* KO could be assessed indirectly based on viability after puromycin selection, which was our subsequent strategy applied to primary human T cells (Figure S1). Indeed, NR2F6 loss resulted in reduced CAR-T cell viability and expansion and consequently failed to improve *in vitro* tumor killing (Figure S2).

### NR2F6 acts as an inducible human transcriptional checkpoint

To elucidate how NR2F6 is induced and maintained under chronic stimulation, we performed promoter and expression analyses (Figure 2). TRANSFAC-based mapping identified multiple putative binding sites for nuclear factor of activated T cells (NFAT), signal transducer and activator of transcription (STAT), and retinoic acid receptor/retinoid X receptor (RAR/RXR) within the human *NR2F6* promoter, indicating that NR2F6 is embedded in core TCR-responsive and related transcriptional circuits (Figure 2A). Consistent with murine data,^25^ reverse transcription-quantitative PCR (RT-qPCR) revealed robust, stimulus-strength-dependent *NR2F6* upregulation after polyclonal TCR activation, peaking at 48 h (Figures 2B and 2C). Repeated TCR engagement every second day via anti-CD3 antibodies maintained elevated *NR2F6* mRNA over at least 8 days, mirroring its sustained expression in TILs (Figure 2D). Luciferase reporter assays performed with the proximal *NR2F6* promoter in Jurkat cells demonstrated that combined co-expression of *RAR* and *RXR* can activate *NR2F6* transcription (Figure 2E). Given that retinoic acid (RA), a physiological RAR/RXR agonist, is abundant within the tumor microenvironment,^26^ these findings suggest that this mechanism may contribute to sustained NR2F6 upregulation during chronic antigen exposure.

**Figure 2:**
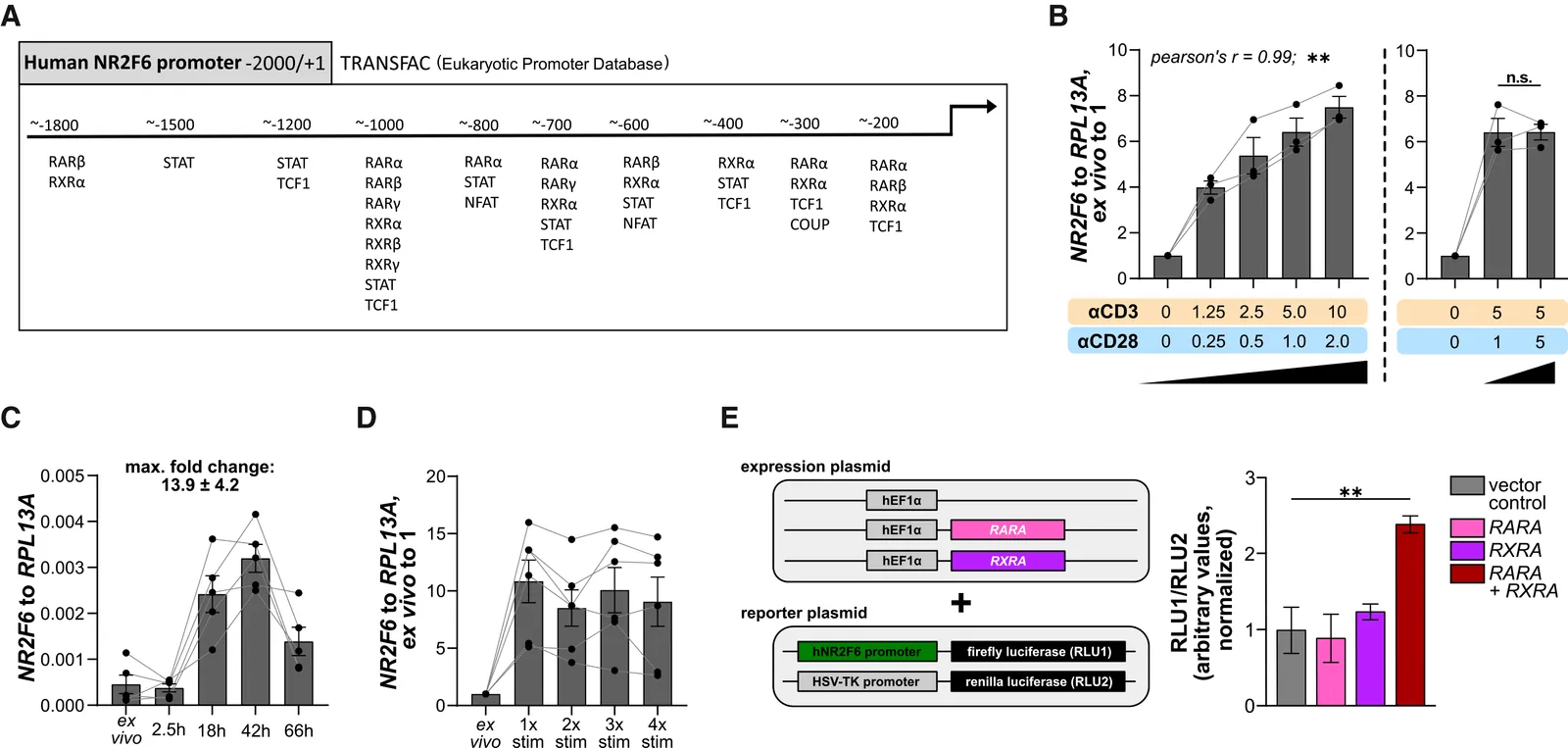
NR2F6 functions as an inducible immune checkpoint that is sustainedly expressed during chronic stimulation *in vitro* (A) Simplified TRANSFAC analysis of the human *NR2F6* promoter showing described and predicted transcription factor binding sites. (B–D) *NR2F6* expression in primary human T cells assessed by RT-qPCR under various stimulation contexts via plate-bound anti-CD3 and soluble anti-CD28 antibodies. (B) Expression 24 h after stimulation with varying concentrations of anti-CD3 and anti-CD28, as specified (*n* = 3 donors, Pearson’s r, log([anti-CD3]) vs. mean *NR2F6* expression, and paired *t* test, respectively). (C) Time-course analysis upon stimulation with 10 μg/mL plate-bound anti-CD3 and 2 μg/mL soluble anti-CD28 antibodies up to 66 h post-stimulation. (*n* = 5 donors). (D) Expression following repeated stimulation with 10 μg/mL plate-bound anti-CD3 and 2 μg/mL soluble anti-CD28 antibodies every 48 h for up to 8 days (*n* = 6 donors). (E) Human *NR2F6* proximal promoter luciferase reporter gene assay. Jurkat-TAg cells were co-transfected with *RARA, RXRA*, or empty vector together with a promoter firefly luciferase reporter (RLU1) and the control reporter vector pTK-Renilla-Luc (RLU2). Constructs (left) together with quantification (right) of the RLU1/RLU2 ratio are shown. (*n* = 5 replicates, unpaired *t* test). All data are represented as mean ± SEM and, where applicable, together with connected individual donor data points. n.s., not significant; ∗*p* < 0.05, ∗∗*p* < 0.01

Together, these findings define NR2F6 as a TCR-inducible transcriptional checkpoint sustained by reinforcing regulatory circuits, providing a mechanistic basis for its persistent upregulation in TILs under chronic antigen exposure in the tumor microenvironment (TME) and enabling NR2F6 to function as a durable brake on human T cell immunity in chronically stimulated environments such as tumors.

### NR2F6 broadly suppresses human T cell activation and effector programs

Having established NR2F6 as an inducible checkpoint in human T cells, we next assessed whether its inhibitory role, originally defined in murine models, is evolutionary conserved in human cells. Using Jurkat reporter cells and primary CD3^+^ T cells with lentiviral *NR2F6* OE or CRISPR-Cas9-mediated KO (Figure S3), we observed that *NR2F6* OE consistently attenuated TCR-driven activation, whereas *NR2F6* loss enhanced it (Figures 3 and S4).

**Figure 3:**
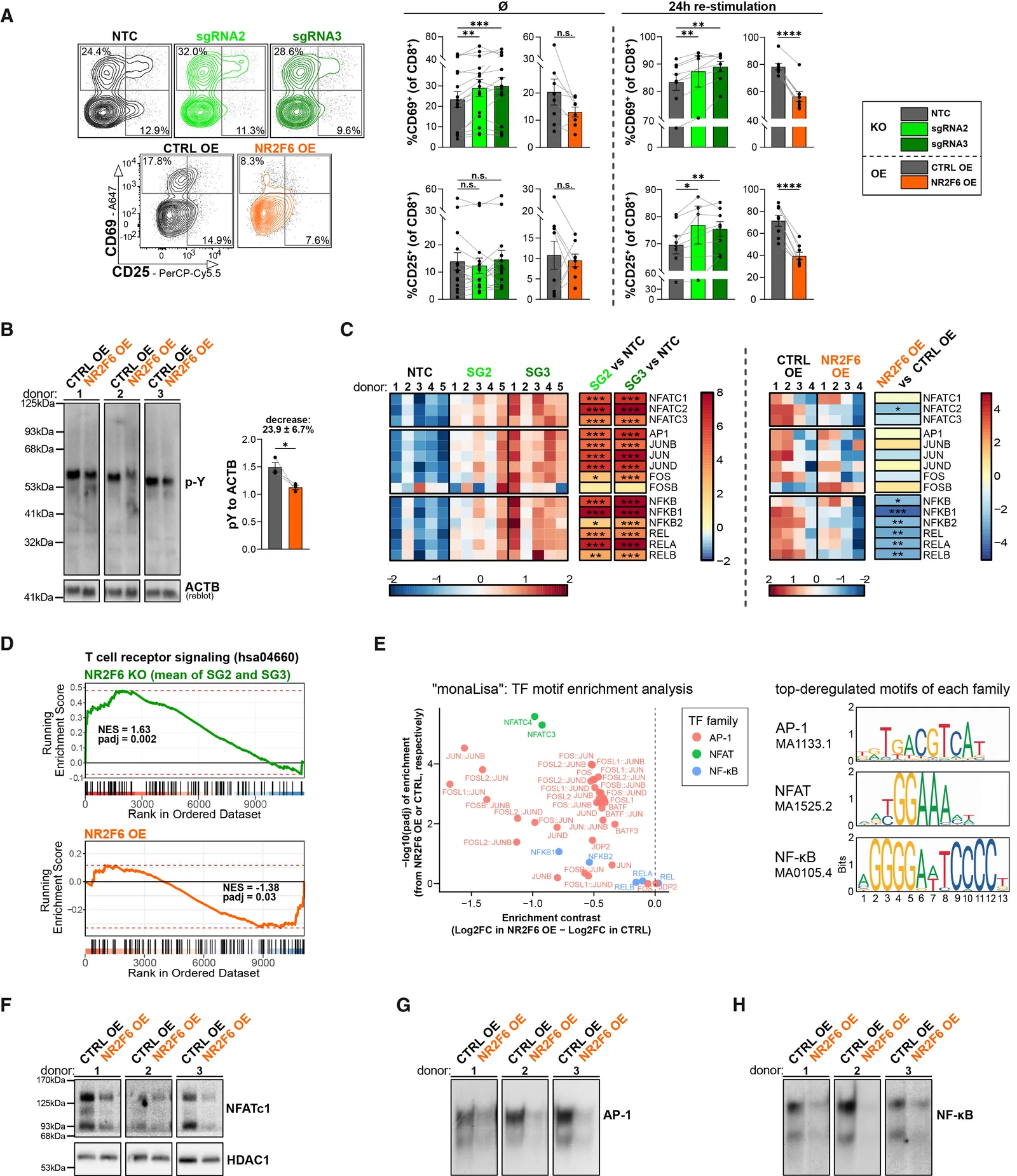
NR2F6 dampens TCR signaling, resulting in reduced T cell activation Stably *NR2F6*-modified human T cells were analyzed upon selection and a 2-week expansion period. *NR2F6*-deficient cells are represented in green, and *NR2F6* OE cells are represented in orange. (A) Flow cytometric characterization of the activation markers CD69 and CD25 (pre-gated on CD8^+^ T cells) in resting cells and upon 24 h re-stimulation via 0.3125 or 5 μg/mL (for KO or OE cells, respectively) plate-bound anti-CD3 antibodies. Representative dot plots together with quantification (mean ± SEM together with connected individual donor data points) are shown (n = 4–15 donors, paired *t* test). (B) Immunoblot of phosphotyrosine (p-Y) upon 24 h re-stimulation via 5 μg/mL plate-bound anti-CD3 antibodies. Immunoblots (left) together with densitometric quantification (right) are shown (*n* = 3 donors, paired *t* test). (C–E) Bulk RNA-seq analysis of rested *NR2F6* KO and 4 h re-stimulated *NR2F6* OE cells. (C) *decoupleR* analysis displaying distribution of inferred T cell activation-related transcription factor activities within groups based on enrichment scores derived from gene regulatory networks (left) and changes in activities between *NR2F6* KO/OE and respective controls together with significance indicated by asterisks (right). (D) Gene set enrichment analysis of the “T cell receptor signaling” KEGG pathway based on ranked gene lists according to (mean) FC of *NR2F6* KO/OE cells compared with their respective controls. Enrichment plots together with normalized enrichment scores (NES) and adjusted *p* values (padj) are shown (*n* = 5 donors for *NR2F6* KO; *n* = 4 donors for *NR2F6* OE). (E) ATAC-seq analysis of sorted CD8^+^ T cells following 4 h re-stimulation with 5 μg/mL plate-bound anti-CD3 antibodies. *MonaLisa* Motif enrichment analysis was performed in differentially accessible peaks. Enrichment contrasts between CTRL and NR2F6 OE cells for transcription factor motifs of the NFAT, AP-1, and NF-κB families are shown, together with the top deregulated motif from each family obtained from the JASPAR database. (*n* = 4 donors). (F–H) Analysis of NFAT, AP-1, and NF-κB activity in nuclear extracts of *NR2F6* OE cells following re-stimulation with anti-CD3 antibodies (*n* = 3 donors each). (F) Immunoblot analysis of nuclear NFATc1 protein levels following 24 h re-stimulation. (G and H) EMSA showing (G) AP-1 and (H) NF-κB DNA-binding activity following 4 h re-stimulation. ∗*p* < 0.05, ∗∗*p* < 0.01, ∗∗∗*p* < 0.001, ∗∗∗∗*p* < 0.0001.

Upon polyclonal restimulation via anti-CD3 antibodies, *NR2F6* OE reduced CD69 and CD25 surface expression, whereas it was enhanced upon KO (Figure 3A). In addition, OE cells displayed decreased tyrosine phosphorylation upon restimulation (Figure 3B). Transcription factor activity inference via *decoupleR*^27^ analysis revealed potent enhancement of NFAT, activator protein 1 (AP-1), and nuclear factor kappa-light-chain-enhancer of activated B cells (NF-κB) activities in *NR2F6* KO cells, again with reciprocal effect in OE cells (Figure 3C). These findings were corroborated by gene set enrichment analysis (GSEA) of the “TCR signaling” Kyoto Encyclopedia of Genes and Genomes (KEGG) pathway, which showed pronounced gene enrichment in KO cells and depletion in OE cells (Figure 3D). These findings were further confirmed across additional molecular layers. Assay for transposase-accessible chromatin using sequencing (ATAC-seq) of *NR2F6* OE cells, combined with *monaLisa*^27^ motif enrichment analysis in differentially accessible genomic regions, showed that NFAT, AP-1, and NF-κB transcription factor binding motifs are stronger enriched in regions that are more accessible in controls. In other words, these regulatory elements become less accessible upon *NR2F6* OE, consistent with reduced activity of the corresponding transcriptional programs (Figure 3E). The most direct confirmation was obtained at the protein level. Upon polyclonal restimulation via anti-CD3 antibodies, *NR2F6* OE cells displayed reduced nuclear accumulation of NFATc1 (Figure 3F), accompanied by diminished binding activity to AP-1 and NF-κB consensus sequences (Figures 3G and 3H). Collectively, these data provide direct evidence that NR2F6 suppresses NFAT-, AP-1-, and NF-κB-mediated signaling pathways.

Functionally, *NR2F6* OE nearly abrogated interleukin-2 (IL-2) and interferon-gamma (IFN-γ) production, both of which have previously been shown to be directly regulated by NR2F6 at their respective promoter regions in murine T cells,^5^ as well as granzyme B (GZMB), a central mediator of T cell cytotoxicity. In contrast, *NR2F6* KO increased these effector outputs. Specifically, *NR2F6* OE markedly reduced intracellular levels of all these cytokines after short-term (4 h) restimulation and suppressed their secretion into culture supernatants upon 24 h stimulation, whereas the opposite effect was observed upon *NR2F6* KO (Figures 4A and 4B). Transcriptomic analysis confirmed broad repression of cytotoxic gene networks, including major components of the cytotoxic machinery (Figure 4C), and GSEA of KEGG pathways highlighted significant deregulation of “Cytokine-Cytokine Receptor Interactions,” “JAK-STAT-”, and “Chemokine-signaling pathways” (Figures 4D and 4E).

**Figure 4:**
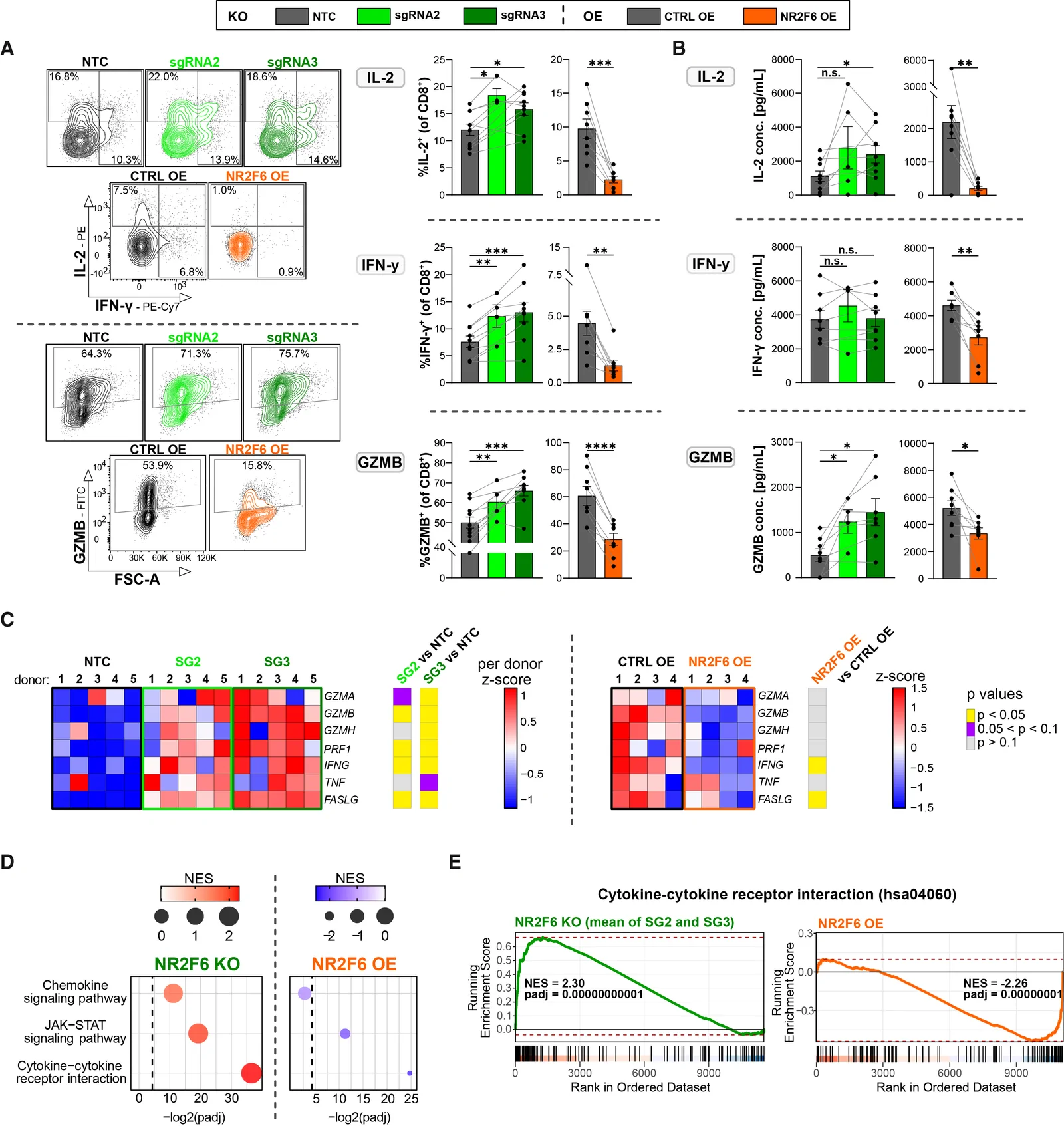
NR2F6 acts as a negative regulator of cytokine expression and effector functions Stably *NR2F6*-modified human T cells were analyzed upon selection and a 2-week expansion period. *NR2F6*-deficient cells are represented in green, and *NR2F6* OE cells are represented in orange. (A and B) Analysis of IL-2, IFN-γ, and GZMB production upon re-stimulation with anti-CD3 antibodies. (A) Intracellular flow cytometry upon 4 h restimulation via 5 μg/mL anti-CD3 antibodies in the presence of brefeldin A and monensin (added 45 min post re-stimulation) pre-gated on CD8^+^ T cells. (*n* = 4–8 donors, paired *t* test). (B) Bioplex analysis after 24 h re-stimulation (0.3125 μg/mL for KO and 5 μg/mL for OE cells) measuring concentration from supernatant. (*n* = 4–8 donors, paired *t* test). Data are represented as mean ± SEM together with connected individual donor data points. (C–E) Bulk RNA-seq analysis of rested *NR2F6* KO and 4 h re-stimulated *NR2F6* OE cells. (C) Heatmap illustrating the variable expression of cytotoxicity-related genes in *NR2F6* KO/OE and respective controls using (donor-specific) z-scores together with *DESeq2*-derived *p* values indicating the significance of change in expression. (D) Gene set enrichment analysis of cytokine (signaling) and chemokine-related KEGG pathways based on a ranked gene list according to the (mean) FC of *NR2F6* KO/OE compared to respective controls. Normalized enrichment score (NES) and adjusted *p* values (padj) are shown. (E) Enrichment plot for the cytokine-cytokine receptor interaction. (*n* = 5 donors for *NR2F6* KO/; *n* = 4 donors for *NR2F6* OE). ∗*p* < 0.05, ∗∗*p* < 0.01, ∗∗∗*p* < 0.001, ∗∗∗∗*p* < 0.0001.

Of note, while *NR2F6* OE exerted a profound inhibitory effect on human T cell activation and effector functions, *NR2F6* deletion produced more modest yet consistent enhancements, likely reflecting *NR2F6* induction prior to gene disruption together with compensatory adaptations during prolonged culture or non-physiological effects of the strong overexpression. Collectively, within these limitations, the data support NR2F6 as a negative regulator of activation, cytokine production, and cytotoxicity-associated programs in human T cells.

### NR2F6 constrains differentiation and modulates TCF-1-associated functional readouts in a context-dependent manner

Because activation status and lineage fate are tightly coupled, we next asked whether NR2F6 also shapes T cell differentiation (Figure 5). Phenotyping of C-C chemokine receptor 7 (CCR7)/CD45RO subsets revealed that *NR2F6* OE favored naive (CCR7^high^ CD45RO^low^) and central memory-like (CCR7^high^ CD45RO^high^) populations while limiting effector memory and terminal effector subsets (CCR7^low^ CD45RO^high^ and CCR7^low^ CD45RO^low^, respectively) (Figure 5A). Consistent with this observation, GSEA demonstrated a significant negative enrichment of KEGG pathways associated with T cell differentiation in *NR2F6* OE cells, whereas the same pathways showed reciprocal positive enrichment upon *NR2F6* deletion (Figure 5B).

**Figure 5:**
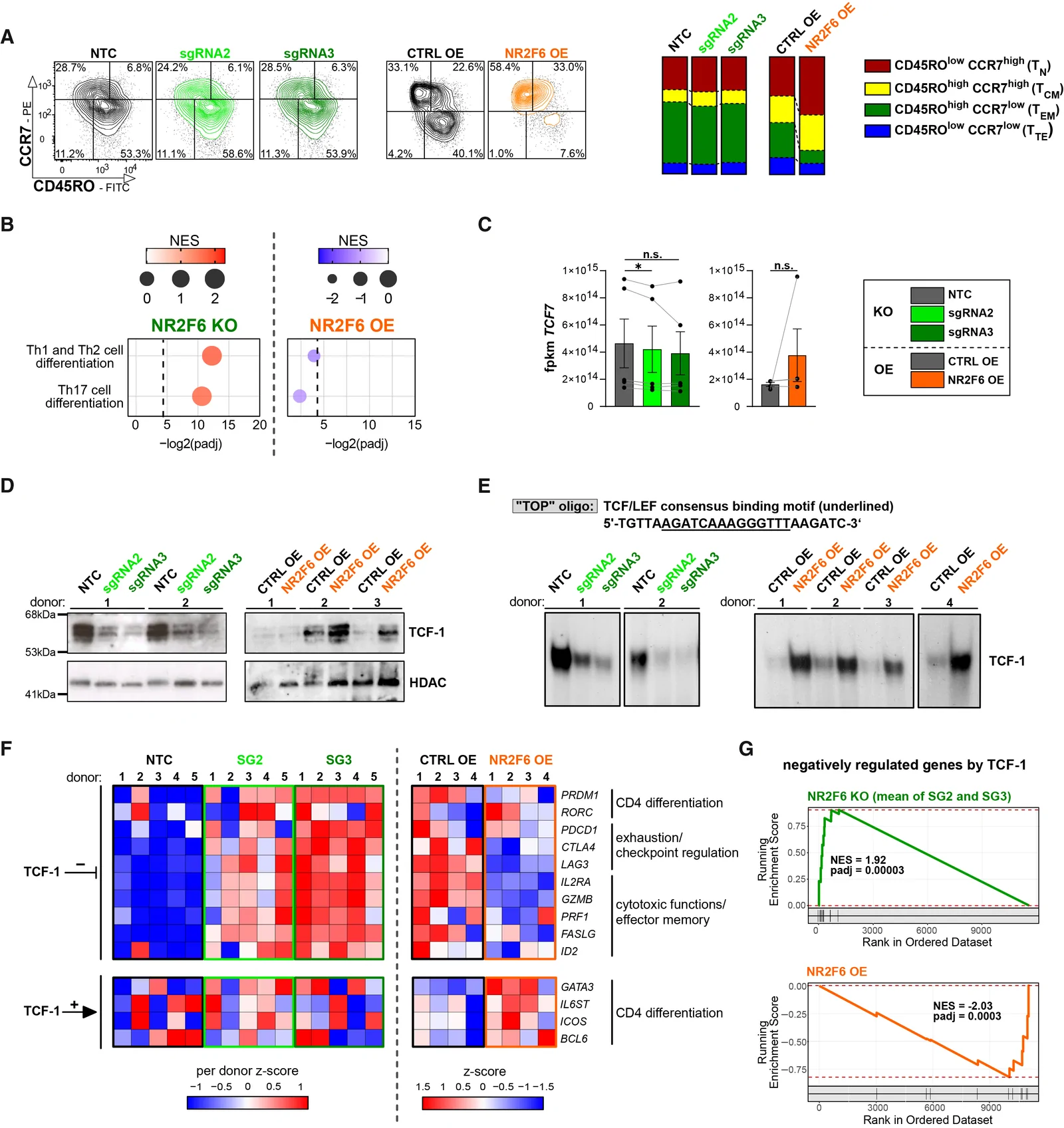
NR2F6 modulates TCF-1-associated differentiation readouts Stably *NR2F6*-modified human T cells were analyzed upon selection and a 2-week expansion period. *NR2F6*-deficient cells are represented in green, and *NR2F6* OE cells are represented in orange. (A) Flow cytometric characterization of the differentiation markers CCR7 and CD45RO (pre-gated on CD8^+^ T cells) and corresponding classification into naive (T_N_), central memory (T_CM_), effector/memory (T_EM_), and terminal effector (T_TE_) T cells. Representative contour plots as well as quantification are shown (*n* = 8–12 donors). (B) Gene set enrichment analysis (GSEA) of T cell differentiation-related KEGG pathways based on a ranked gene list according to the (mean) FC of *NR2F6* KO/OE compared to respective controls. Normalized enrichment score (NES) and adjusted *p* value (padj) are shown. (C) FPKM-normalized expression levels of *TCF7.* (mean ± SEM together with connected individual donor data points). (D and E) Analysis of TCF-1 after 24 h re-stimulation with anti-CD3 antibodies (at a concentration of 0.3125 μg/mL or 5 μg/mL for KO or OE cells, respectively). (D) Immunoblot of nuclear TCF-1 protein levels as well as (E) EMSA showing TCF/LEF binding (*n* = 2–4 donors). (F) Heatmap illustrating the variable expression of TCF-1-regulated genes in *NR2F6* KO/OE and respective controls using (donor-specific) z-scores. (G) GSEA and the corresponding enrichment plot of negatively regulated genes by TCF-1 based on a ranked gene list according to the (mean) FC of *NR2F6* KO/OE compared to respective controls with NES and adjusted *p* values (padj). Bulk RNA-seq analyses (B, C, F, and G) were performed on rested *NR2F6* KO and 4 h re-stimulated *NR2F6* OE cells. (*n* = 5 donors for *NR2F6* KO; *n* = 4 donors for *NR2F6* OE).

Focusing on TCF-1 (*TCF7*) as a central fate regulator, we found modest but consistent NR2F6-dependent changes at the *TCF7* transcript level that translated into pronounced shifts in nuclear TCF-1 protein abundance and DNA-binding activity at TCF/LEF consensus sites (Figures 5C–5E). Consistent across all levels, *NR2F6* OE enhanced TCF-1 activity, whereas *NR2F6* KO reduced it. Correspondingly, several established TCF-1 target genes (reviewed by Zhao et al.^28^) were significantly deregulated, with particularly strong effects on genes negatively regulated by TCF-1 as evident from GSEA (Figures 5F and 5G).

To further investigate the relationship between NR2F6 and TCF-1, we employed a Jurkat human leukemic T cell line model system. Electrophoretic mobility shift assays (EMSAs) showed that *NR2F6* OE strongly repressed endogenous TCF DNA-binding activity (Figures S5A and S5B), in contrast to the enhanced TCF-1 activity observed in primary human T cells. These findings suggest that NR2F6 modulates TCF-1-associated programs in a context-dependent manner. Consistent with a functional interaction, *NR2F6* OE significantly suppressed the activity of a TCF response element-driven luciferase reporter (Figure S5C), in line with the established transcriptional repressor function of NR2F6 at minimal promoter elements. Together, these data support a context-dependent modulation of TCF-responsive readouts by NR2F6 but do not establish direct NR2F6-TCF-1 binding or direct regulation of TCF-1, as these effects could also arise indirectly through NFAT or other upstream pathways.

## Discussion

The identification of novel immuno-oncological targets is a central goal in cancer immunotherapy. This study provides the first comprehensive characterization of NR2F6 function in primary human T cells and shows that its role as a negative regulator of T cell immunity is evolutionarily conserved and functionally non-redundant, in line with observations in murine models.

Using genetic perturbation in primary human T cells and an investigational CAR-T cell model, we show that NR2F6 acts as a T cell-intrinsic immune checkpoint that coordinately dampens TCR signaling, effector functions, and differentiation. Consistent with prior work in murine model systems, NR2F6 antagonizes NFAT- and AP-1-transcriptional modules. Extending these findings, our transcriptomic, epigenomic, and biochemical analyses also implicate deregulation of the NF-κB pathway as an additional mechanism of NR2F6-mediated immune suppression. Together with the observed alterations in cytokine, chemokine, and cytotoxic effector programs, these data position NR2F6 as a key transcriptional checkpoint governing human T cell effector function.

Several aspects of our experimental system warrant consideration. First, *NR2F6* deletion was achieved through lentiviral delivery of Cas9 and sgRNA, followed by antibiotic selection, enabling robust modulation and functional assessment but also introducing constraints. Because *NR2F6* is rapidly induced upon T cell activation, initial expression preceding gene disruption may partially obscure phenotypes. Moreover, prolonged culture may permit compensatory adaptations. Together with the relatively modest induction of NR2F6 *in vitro* and the viability issues resulting from its long-term loss, these factors likely contribute to the more modest effects observed in the KO compared with the overexpression setting. Conversely, enforced overexpression may not fully recapitulate physiological regulation. However, *NR2F6* has been reported to be strongly upregulated in TILs,^6^ supporting the biological relevance of this model, although broader validation across cohorts and cancer types remains necessary. Supporting the functional significance of our OE data, even modest upregulation of endogenous *NR2F6* by CRISPR activation (∼1.4-fold at the transcript level) was associated with a detectable trend toward reduced T cell activation and effector cytokine production (Figure S6).

While our analyses focused primarily on effector functions classically associated with CD8^+^ T cells, comparable NR2F6-dependent effects were observed in CD4^+^ T cells (Figure S7), indicating that NR2F6 broadly regulates both CD4^+^ and CD8^+^ subsets. This is important to highlight given the growing recognition of CD4^+^ T cells as key contributors to long-term anti-tumor immunity.^29^^,^^30^^,^^31^ Consequently, NR2F6’s effects on CD4^+^ T cell differentiation and functional states warrant more detailed investigation in future studies.

Another remaining limitation is that the orphan receptor status of NR2F6 is unresolved. Identifying ligands or microenvironmental cues that govern NR2F6 activity will be essential to understand how this pathway is engaged in tumor-mediated immune evasion and to evaluate its therapeutic tractability.

A major conceptual advance emerging from our data is that NR2F6 is not only induced by TCR engagement but can also be maintained through reinforcing regulatory circuits. Promoter mapping and reporter assays revealed that RAR/RXR can directly activate the *NR2F6* promoter, providing a potential mechanistic explanation for its persistent upregulation under chronic antigen exposure and in human TILs within RA-high tumor microenvironments.^26^ However, our simplified *in vitro* system did not account for ligand availability for RAR/RXR, and this hypothesis requires testing in more physiologically relevant settings. Nevertheless, such reinforcing behavior may help explain how TME cues establish *NR2F6* expression, which is then sustained even when upstream stimuli fluctuate, thereby stabilizing dysfunctional states in chronically stimulated T cells.

Our CAR-T cell data underscore the functional impact of this checkpoint. Enforced *NR2F6* expression, modeling its high levels in TILs, markedly impairs CAR-T cell activation, cytokine production, and serial killing under repeated tumor challenge, whereas NR2F6 loss did not improve tumor killing and was associated with reduced viability and expansion. These effects may reflect restimulation-induced cell death, reduced fitness, or model-specific factors, none of which was directly tested. These findings suggest that NR2F6 helps set a threshold between productive effector responses and overstimulation-induced attrition and highlight the need for carefully tuned *NR2F6* targeting strategies in engineered T cell therapies. Notably, clinical profiling of anti-CD19 CAR-T cells has shown higher *NR2F6* expression in non-responders, further supporting the notion that NR2F6 restricts CAR-T cell function.^32^ Collectively, these observations argue for *in vivo* validation and extension of our findings. Humanized mouse models engrafted with tumors, coupled with longitudinal flow cytometric analyses, will be essential to capture additional NR2F6-dependent processes, such as migration, differentiation, and the development of *bona fide* T cell exhaustion, which cannot be fully recapitulated *in vitro*. Given the pronounced deregulation of cytokine and chemokine-related pathways upon *NR2F6* loss, such models may also reveal enhanced tumor infiltration, as previously observed in murine systems.^5^^,^^6^ The magnitude of NR2F6 KO effects *in vivo*, cannot be inferred from the present data. It is, however, even conceivable that the beneficial impact of *NR2F6* KO will be more apparent *in vivo*, as the tumor microenvironment is expected to induce *NR2F6* expression in the control CAR-T cell group, while survival or fitness defects observed *in vitro* may be mitigated under physiological conditions.

Mechanistically, NR2F6 perturbation was associated with context-dependent changes in TCF-1-related readouts. Of note, already prior murine studies in thymocytes and a CAR-T cell model^9^^,^^33^ demonstrated a functional connection between NR2F6 and TCF-1, supporting a relationship between these transcription factors. Similarly, our data in human T cells support a functional connection between NR2F6 and TCF-1-associated transcriptional programs, although they do not fully define the underlying molecular mechanism. We demonstrate modulation of a TCF response element by NR2F6, although the direction of this effect is highly context dependent. Of particular note, although our Jurkat luciferase assays show modulation of a TCF response element, these experiments do not demonstrate direct NR2F6-TCF-1 interaction or direct regulation of TCF-1. The effects observed on TCF-1 and its transcriptional regulation in primary human T cells may still represent a secondary consequence of sustained NR2F6-mediated NFAT repression, a well-described regulatory link that has been previously reviewed, for example, by Seo et al.^34^ Thus, future studies will be required to explicitly determine whether NR2F6 can also directly modulate TCF-1 function in primary T cells and, if so, whether this is mediated primarily through changes in chromatin accessibility, co-factor landscapes, direct protein interactions, or indirect modulation of upstream signaling.

In summary, our findings establish NR2F6 as a signal-integrating transcriptional checkpoint in human T cells that restrains TCR-driven transcriptional and effector programs, thereby promoting acute T cell dysfunction *in vitro*.

### Limitations of the study

This study has some limitations that should be considered when interpreting the data. All experiments were performed *in vitro* and therefore do not capture the full complexity of tumor-associated antigen exposure, tissue-specific microenvironmental cues, CAR-T cell persistence, or long-term differentiation trajectories *in vivo*. As a result, the present work demonstrates acute dysfunction under controlled stimulation but does not formally address *bona fide* exhaustion or establish *in vivo* relevance. Second, *NR2F6* modulation relied on lentiviral strategies in activated, cultured T cells. This workflow entails early *NR2F6* induction and allows compensatory adaptations, which likely contribute to the more modest loss-of-function phenotypes compared with overexpression. Conversely, enforced overexpression may exceed physiological levels and is best viewed as a model of elevated NR2F6 activity rather than a full representation of endogenous regulation, although complementary CRISPRa experiments support a biological impact of even modest endogenous NR2F6 upregulation. Third, the mechanistic link between NR2F6 and TCF-1 is not yet defined at the molecular level. The TCF-1-associated findings do not demonstrate a direct NR2F6-TCF-1 interaction; indirect regulation through upstream pathways remains possible. Finally, NR2F6 remains an orphan nuclear receptor, and the physiological ligands and microenvironmental signals that govern its activity in human tumors are still unknown, representing an important focus for future investigation.

## Resource availability

### Lead contact

Requests for further information and resources should be directed to and will be fulfilled by the lead contact, Gottfried Baier (gottfried.baier@i-med.ac.at).

### Materials availability

All unique/stable reagents generated in this study are available from the lead contact without restriction.

### Data and code availability

• Bulk RNA-seq raw data generated in this study have been deposited in the Gene Expression Omnibus (GEO) under accession number GSE318203 and are publicly available as of the date of publication.
• Bulk ATAC-seq raw data (aligned bam files) generated in this study have been deposited in the Gene Expression Omnibus (GEO) under accession number GSE336068 and are publicly available as of the date of publication.
• All other raw data are available from the lead contact upon reasonable request.
• This paper does not report any original code. Publicly available codes used for analysis are specified in the STAR Methods section.
• Any additional information required to reanalyze the data reported in this paper is available from the lead contact upon request.

## Acknowledgments

We thank Viana Wille, Nina Posch, and Dr. Friedrich Fresser (Institute of Cell Genetics, Medical University of Innsbruck) for excellent technical assistance. We would like to acknowledge all funding agencies enabling this work; these are in part or fully: the Austrian Science Fund (FWF; grant-10.13039/100000201DOI 10.55776/P31383 to G.B., grant-10.13039/100000201DOI 10.55776/P34368 to K.S., grant-DOI 10.55776/PAT9292223 to T.G., and grant-DOI 10.55776/T1292 to V.K.); the DOC fellowship from the 10.13039/501100001822Austrian Academy of Sciences (ÖAW) to J.K.; and the 10.13039/501100000781European Research Council (ADG 786462, PoC 101054365, and PoC 101189004, to G.B.).

## Author contributions

Conceptualization and design: J.K. and G.B. designed the study. G.B. supervised the project. Experiments and analysis: J.K. conceived, planned, performed, and analyzed all experiments with support as follows: D.F. assisted with generation and analysis of lentiviral *NR2F6* knockout experiments (Figures 3, 4, 5, S2, and S3); T.G. performed nuclear TCF-1, AP-1, and NF-κB immunoblotting/electrophoretic mobility shift assays (Figures 3G, 3H, 5D, and 5E); N.Th. performed Bioplex cytokine analysis and NFAT immunoblotting (Figures 1G, 2B, 3F, and S2H); D.S. assisted with *NR2F6* expression analyses (Figures 2C and 2D); I.H. assisted with the serial killing assay (Figure 1F); V.L. performed *in vitro* Cas9 cleavage validation assays and assisted with generation of lentiviral *NR2F6* OE experiments for revision (Figures 3F–3H and S1B); N.To. assisted with generation and analysis of lentiviral *NR2F6* OE experiments (Figures 1, 3, 4, and 5); T.S. assisted with TRANSFAC promoter analysis and Jurkat luciferase assays (Figures 2A and 2E); and K.S. assisted with *NR2F6* expression analysis (Figure 2C) and sgRNA validation in HEK293T cells (Figures S1C and S1D). Manuscript preparation and approval: J.K. visualized the data and generated the figures. J.K. and G.B. wrote the original draft of the manuscript. All authors read, critically revised, and approved the final manuscript.

## Declaration of interests

The authors declare the following competing interests: V.K., J.K., K.S., D.H., D.W., and G.B. are inventors on patents related to immunological targets in the field of immuno-oncology.

## Declaration of generative AI and AI-assisted technologies in the manuscript preparation process

During the preparation of this work, the authors used Perplexity and ChatGPT5.0 in order to improve phrasing. After using these tools, the authors reviewed and edited the content as needed and take full responsibility for the content of the publication.

## STAR★Methods

### Key resources table

**Table:** 

REAGENT or RESOURCE	SOURCE	IDENTIFIER
**Antibodies**		

Alexa Fluor® 488 anti-human CD3 Antibody (clone UCHT1)	BioLegend	RRID: AB_389310; Cat#300415
Alexa Fluor® 488 anti-human CD45RO Antibody (clone UCHL1)	BioLegend	RRID: AB_528823; Cat#304212
Alexa Fluor® 647 anti-human CD69 Antibody (clone FN50)	BioLegend	RRID: AB_528871; Cat#310918
Brilliant Violet 510™ anti-human CD8a Antibody (clone RPA-T8)	BioLegend	RRID: AB_2561942; Cat#301048
FITC anti-human/mouse Granzyme B Antibody (clone GB11)	BioLegend	RRID: AB_2114575; Cat#515403
InVivoMAb anti-human CD3 (clone OKT3)	BioXcell	RRID: AB_1107632; Cat#BE0001-2
InVivoMAb anti-human CD28 (clone 9.3)	BioXcell	RRID: AB_2687729; Cat#BE0248
Mouse monoclonal anti-HDAC1 (clone 10E2)	Santa Cruz	RRID: AB_2118083; Cat#sc-81598
Mouse monoclonal anti-NFATc1 (clone 7A6)	Santa Cruz	RRID: AB_2152503; Cat#sc-7294
Mouse monoclonal anti-NR2F6 (clone 2H2B8)	ProteinTech	RRID: AB_2155653; Cat#60117-2-Ig
Mouse monoclonal anti-phospho-tyrosine (clone 4G10)	Cell Signaling	RRID: AB_3096295; Cat#96215
Mouse monoclonal anti-TCF-1 (clone A-79)	Santa Cruz	RRID: AB_1130027; Cat#sc-101170
Mouse monoclonal anti-β-actin (clone C-11)	Santa Cruz	RRID: AB_630835; Cat#sc-1615
Pacific Blue™ anti-human CD4 Antibody (clone OKT4)	BioLegend	RRID: AB_571953; Cat#317424
PE anti-human CD279 (PD-1) Antibody (clone EH12.2H7)	BioLegend	RRID: AB_940483; Cat#329906
PE anti-human IL-2 Antibody (clone MQ1-17H12)	BioLegend	RRID: AB_315094; Cat#500307
PE/Cyanine7 anti-human CD223 (LAG-3) Antibody (clone 11C3C65)	BioLegend	RRID: AB_2629753; Cat#369310
PE/Cyanine7 anti-human CD45RA Antibody (clone HI100)	BioLegend	RRID: AB_10708879; Cat#304126
PE/Cyanine7 anti-human IFN-γ Antibody (clone 4S.B3)	BioLegend	RRID: AB_2123323; Cat#502528
PerCP/Cyanine5.5 anti-human CD25 Antibody (clone M-A251)	BioLegend	RRID: AB_2561979; Cat#356112
PerCP/Cyanine5.5 anti-human CD366 (Tim-3) Antibody (clone F38-2E2)	BioLegend	RRID: AB_2561934; Cat#345016

**Biological samples**		

Leukocyte reduction cones/PBMCs of healthy donors	blood bank of Innsbruck University Hospital	N/A

**Chemicals, peptides, and recombinant proteins**		

Acridine Orange/Propidium Iodide stain	Biocat	Cat#F23001-LG
BD GolgiPlug™ Protein transport inhibitor (Containing Brefeldin A)	BD Biosciences	Cat#555029
BD GolgiStop™ Protein transport inhibitor (Containing Monensin)	BD Biosciences	Cat#554724
Biotinylated recombinant EGFR	Creative Biolabs	Cat#CARD-LX025
Bovine serum albumin (BSA)	Sigma	Cat#A7906
DMEM	PAN Biotech	Cat#P04-03500
dNTPs	Biozym	Cat#331520
EMSA stabilizing solution D	Activ Motif	Cat#37488
Ethylenediaminetetraacetic acid (EDTA)	Thermo Fisher Scientific	Cat#J15694.AP
Fetal calf serum	Biowest	Cat#S1810-500
Fixation Buffer	BioLegend	Cat#420801
GoTaq G2 Hot Start Polymerase	Promega	Cat#M7401
Halt™ Protease and Phosphatase Inhibitor Cocktail	Thermo Fisher Scientific	Cat#78444
HBSS	PAN Biotech	Cat#P04-34500
Human recombinant IL-2	BioLegend	Cat#589102
Intracellular Staining Permeabilization Wash Buffer (10X)	BioLegend	Cat#421002
Ionomycin	Sigma	Cat#I0634
L-Glutamine	PAN Biotech	Cat#P04-80050
Lenti-X™ Concentrator	Takara	Cat#631232
Lipofectamine 2000	Thermo Fisher Scientific	Cat#11668019
Lymphopure	BioLegend	Cat#426202
MgCl_2_	Promega	Cat#A351H
Non-essential amino acids	Sigma	Cat#M7145
PenStrep	PAN Biotech	Cat#P06-07100
peqGREEN DNA dye	Peqlab	Cat#732–2960
Phosphate buffered saline (PBS)	Thermo Fisher Scientific	Cat#J62036.K3
Phorbol 12,13-dibutyrate	Sigma	Cat#P1269
Polybrene	Sigma	Cat#TR-1003-G
Puromycin	Sigma	Cat#P8833
recombinant *S. pyogenes* Cas9 nuclease	IDT	Cat#1081059
RIPA buffer	Sigma	Cat#R02789
RPMI 1640	PAN Biotech	Cat#P04-17500
SB415286	Selleckchem	Cat#S2729
Sodium pyruvate	Sigma	Cat#S8636

**Critical commercial assays**		

Anti-Mouse Detection Module	ProteinSimple	Cat#DM-002
12–230 kDa Separation Module	ProteinSimple	Cat#SM-W001
Bicinchoninic acid (BCA) assay	Thermo Fisher Scientific	Cat#23227
Bio-Plex Pro Human Cytokine IL-2 Set	Biorad	Cat#171-B5003M
Bio-Plex Pro Human Cytokine IFN-γ Set	Biorad	Cat#171-B5019M
Cell Line Nucleofector™ Kit	Lonza	Cat#VCA-1003
Dual-Luciferase Reporter Assay	Promega	Cat#E1960
Guide-it™ sgRNA Screening Kit	Takara Bio	Cat#632639
Granzyme B Human ProcartaPlex™ Simplex Kit	Thermo Fisher Scientific	Cat#EPX01A-12027–901
LS columns and a QuadroMACS separator	Miltenyi Biotec	Cat#130–042–401 and 130–091–051
MojoSortTM Human CD3Cell Isolation Kit	BioLegend	Cat#480131
Monarch PCR & DNA Cleanup Kit	New England Biolabs	Cat#T1030L
NucleoSpin Tissue Kit	Macherey-Nagel	Cat#740952
Omniscript RT Kit	Qiagen	Cat#205111
RNeasy Kit	Qiagen	Cat#74016

**Deposited data**		

BulkRNA-Seq raw data of *NR2F6*-deficient or *NR2F6*-overexpressing primary human T cells, genetically modified by lentiviral transduction (CRISPR-Cas9 or direct *NR2F6* integration) followed by puromycin selection and 15-day expansion. 4 h polyclonal re-stimulation via 5 μg/mL anti-CD3 antibodies in case of *NR2F6*-OE	This Paper	deposited in the Gene Expression Omnibus (GEO) under accession number GSE318203
ATAC-Seq profiling on *NR2F6*-overexpressing primary human T cells generated via lentiviral transduction with direct *NR2F6* integration, followed by puromycin selection and 15 days of expansion, after which cells were CD8-sorted and subjected to 4 h of polyclonal restimulation using 5 μg/mL anti-CD3 antibodies.	This Paper	deposited in the Gene Expression Omnibus (GEO) under accession number GSE336068

**Experimental models: Cell lines**		

A549^*B2M*−/−^	Abcam	RRID: CVCL_C3CC; Cat#ab286573
A549^*B2M*−/− eGFP^	This paper	N/A
A549^*B2M*−/− mCherry^	This paper	N/A
HEK293T	Abcam	RRID: CVCL_0063; Cat#ab255449
Jurkat-TAg T cells	kind gift from G.R. Crabtree; Stanford University, CA	N/A
Jurkat^NTC^	This paper	N/A
Jurkat^*NR2F6−*/–(sgRNA2)^	This paper	N/A
Jurkat^*NR2F6−*/–(sgRNA3)^	This paper	N/A
Jurkat^CTRL−OE^	This paper	N/A
Jurkat^*NR2F6*−OE^	This paper	N/A
Jurkat^dCas9−VPR^	This paper	N/A

**Oligonucleotides**		

CRISPRa: Control crRNA pool – hypothetical target sequences: 5′-GATACGTCGGTACCGGACCG-3′ 5′-GTAACGCGAACTACGCGGGT-3′ 5′-GTCGACGTTATTGCCGGTCG-3′ 5′-GGAAACCTACGTCGACGAAT-3′	Dharmacon	Cat#U-009500–10–20
CRISPRa: *NR2F6* Promoter-targeting crRNA pool – target sequences: 5′-GGCCGCCCTTATAAGGGCAT-3′ 5′-CAGGGGTTAAGGCCCGAGCT-3′ 5′-TTAAGGCCCGAGCTGGGCGA-3′ 5′-GAGGAAGCAAACACAATCTA-3′	Dharmacon	Cat#P-003423-01-0020
CRISPR KO: Control sgRNA (safe harbor, *PPP1R12C, intron 1*) – targeting sequence: 5′-CGTTGCCAGTCTCGATCCGC-3′	GeneScript	custom-synthesized
CRISPR KO: *NR2F6*-targeting sgRNAs – target sequences: sgRNA1: 5′-TACGGTGTCTTCACCTGCGA-3′ sgRNA2: 5′-GTACTGCGCCCGCACATACT-3′ sgRNA:3 5′-AGGTGTAGCTGAGGTTGCGG-3′	Dharmacon	Cat#SG-003423–01/02/03
CRISPR KO: PCR-primer for amplification of *NR2F6* exon 1 – F: 5′-ACGAACGGCGTGGACAAG-3′ R: 5′-CCAAGTTCCACGTTCGATTCC-3′; amplicon spans sgRNA1/sgRNA3 cutting sites.	Eurofins	custom-synthesized
CRISPR KO: PCR-primer for amplification of *NR2F6* exon 3 – F: 5′-CGATCCTCCCATTAGCCTTC-3′ R: 5′-TTCCCTGTCCCTTGAGGTCT-3′; amplicon spans sgRNA2 cutting site.	Eurofins	custom-synthesized
EMSA AP-1 consensus binding site oligonucleotide 5′-CGCTTGATGACTCAGCCGGAA-3′	Eurofins	custom-synthesized
EMSA NF-κB consensus binding site oligonucleotide 5′-CTGGGGACTTTCCGCT-3′	Eurofins	custom-synthesized
EMSA TCF/LEF consensus binding site oligonucleotide (TOP) 5′-TGTTAAGATCAAAGGGTTTAAGATC-3′	Eurofins	custom-synthesized
EMSA mutated TCF/LEF consensus binding site oligonucleotide (FOP) 5′-TGTTAAGAAAAAAGGGTTTAAGATC-3′	Eurofins	custom-synthesized
TaqMan Gene Expression primer & probe for RPL13A	Thermo Fisher/Applied Biosciences	Assay ID: Hs04194366_g1_VIC_MGB_PL
TaqMan Gene Expression primer & probe for NR2F6	Thermo Fisher/Applied Biosciences	Assay ID: Hs00172870_m1_FAM_MGB

**Recombinant DNA**		

hEF1a-PuroR:T2A:Cas9-U6-sgRNA(NTC) lentiviral plasmid	Horizon Discovery	Cat#GSGC11963
hEF1a-PuroR:T2A:Cas9-U6-sgRNA2(NR2F6) lentiviral plasmid	Horizon Discovery	Cat#GSGH11935-247640844
hEF1a-PuroR:T2A:Cas9-U6-sgRNA3(NR2F6) lentiviral plasmid	Horizon Discovery	Cat#GSGH11935-247620068
LentiArt™ Virus Packaging Plasmids	Creative Biolabs	Cat#CART-027CL
lenti-EF1a-dCas9-VPR-Puro	gift from Kristen Brennand	Addgene#99373
NR2F6-Promoter (bp −2145 to −1, relative to TSS)-firefly luciferase reporter plasmid	This paper	N/A
pCDCAR1- anti-EGFR-CAR(Cetuximab) h(41BB-CD3ζ) lentiviral plasmid	Creative Biolabs	Cat#CAR-MZ007
pCDH-CMV-mCherry-T2A-puro lentiviral plasmid	gift from K.Oka	Addgene#72264
pEF1a-(empty)-Neo mammalian expression plasmid	This paper	N/A
pEF1a-RARA-Neo mammalian expression plasmid	This paper	N/A
pEF1a-RXRA-Neo mammalian expression plasmid	This paper	N/A
pEF1a-NR2F6-Neo mammalian expression plasmid	This paper	N/A
pEF1a-NR2F6^C1^^1^^1S^-Neo mammalian expression plasmid	This paper	N/A
pEF1a-TCF7^isoform4^-Neo mammalian expression plasmid	This paper	N/A
pGL4.49[*luc2P*/TCF-LEF/Hygro] reporter plasmid	Promega	Cat#9PIE461
pLV[Exp]-EF1A>ORF Stuffer:IRES:Puro lentiviral plasmid	This paper, VectorBuilder	Vector ID: VB241126-1331hgg
pLV[Exp]-EF1A>hNR2F6[NM_005234.4]:IRES:Puro lentiviral plasmid	This paper, VectorBuilder	Vector ID: VB241127-1225nsn
pLVX-EGFP-IRES-Puro lentiviral plasmid	gift from R. Sobol, described by Fang et al.^35^	Addgene#128652

**Software and algorithms**		

*decoupleR* (v2.8.0)	Badia et al.^36^	N/A
*DESeq2* (v1.42.1)	Love et al.^37^	N/A
FlowJo (v10.7.2.)	BD Biosciences	N/A
GraphPad Prism (v10.2.3)	GraphPad Software, LLC	N/A
Incucyte2022B analysis software	Sartorius	N/A
*monaLisa* (v.1.16.0)	Machlab et al.^27^	N/A
R (v4.3.3.)	R Foundation for Statistical Computing	N/A

**Other**		

BTX T820 Electro Square Porator	ITC Biotech	N/A
DS-11 spectrophotometer	DeNovix	N/A
FACSCanto II (4-2-2 configuration)	BD Biosciences	N/A
GelDoc Go Gel Imaging System	Bio-Rad	N/A
Jess Automated Western System	Proteinsimple	N/A
PHERAstar FS plate reader	BMG LABTECH	N/A
Thermo Neon™ NxT Electroporator	Thermo Fisher Scientific	N/A

### Experimental model and study participant details

#### Primary cell cultures

Primary human T cells were obtained from healthy donors via the Blood Bank of Innsbruck University Hospital as described in the method details section. The cells were derived from left-over material from routine blood donations, and all donors had provided informed consent for the use of residual material for research purposes. No identifiable patient or donor data were provided. Accordingly, and in line with institutional policy, no additional ethics approval was required. Full anonymization precluded consideration of sex or age, which limits assessment of sex-related effects, though donors represent an unsystematic, randomized sample. In total, samples from 35 different donors were investigated, with the number of donors used for each experiment specified in the respective figure legends. No allocation to experimental groups was performed, as the study used *ex vivo* primary cells for mechanistic analyses, with comparisons performed within donors only.

The cells were cultured in RPMI 1640 (PAN Biotech, P04-17500) supplemented with 10% heat-inactivated FBS (Biowest, S1810-500), 100 U/mL penicillin/100 μg/mL streptomycin (PAN Biotech, P06-07100), 2 mM L-glutamine (PAN Biotech, P04-80050), 1 mM sodium pyruvate (Sigma, S8636), and 1× non-essential amino acids (Sigma, M7145) (cRPMI).

#### Cell lines

Jurkat-TAg cells (gift from G.R. Crabtree, Stanford University) were cultured in cRPMI. This cell line originates from a human CD4^+^ T-ALL derived from a 14-year-old male patient. However, since used primarily for method validation and to support primary T cell findings, this does not constrain the study’s generalizability.

A549^*B2M*−/−^ cells (Abcam, ab286573) were cultured in Ham’s F-12K (Kaighn’s) medium (Thermo Fisher Scientific, 21127030) supplemented with 10% FBS, 100 U/mL penicillin & 100 μg/mL streptomycin (from here on F12K++). Derivatives expressing either EGFP or mCherry were generated by stable transduction with pLVX-EGFP-IRES-puro (gift from R. Sobol, Addgene #128652, described by Fang et al.^36^) or pCDH-CMV-mCherry-T2A-puro (gift from K. Oka, Addgene #72264), respectively. A549 cells originate from a human lung adenocarcinoma tumor derived from a 58-year-old male patient. Since these cells were used solely as tumor target cells and were not the primary focus of the study, the potential influence of their specific background was considered negligible.

During this study, the cell lines were not authenticated or tested for mycoplasma contamination.

### Method details

#### Cell counting

Cell numbers and viability were assessed using a LUNA automated cell counter (Logos Biosystems) with acridine orange/propidium iodide staining (Biocat, F23001-LG).

#### T cell isolation

Peripheral blood mononuclear cells (PBMCs) were isolated from leukocyte reduction cones (LRCs) of healthy donors by density centrifugation. LRCs were flushed with HBSS (PAN Biotech, P04-34500) supplemented with 2 mM EDTA, layered over Lymphopure solution (BioLegend, 426202), and centrifuged at 800 rcf for 30 min at room temperature (RT) without brake. PBMCs were collected, washed in HBSS/2 mM EDTA, and pan-T cells were purified by negative selection using the MojoSort Human CD3 Isolation Kit (BioLegend, 480131) in combination with LS columns and a QuadroMACS separator (Miltenyi Biotec, 130–042–401 and 130–091–051) based on the manufacturers’ instruction. As indicated in the individual experimental set-ups, optionally CD8^+^ T cells were additionally purified from pan T cells analogously by negative selection using the MojoSort Human CD8 Isolation Kit (BioLegend, 480129).

#### *NR2F6* promoter and TCF/LEF response element luciferase assay

Jurkat-TAg cells were transiently transfected by electroporation using a BTX T820 Electro Square Porator (ITC Biotech) under optimized conditions (1 × 10^7^ cells in 200 μL RPMI, 450 V/cm, five 99 ms pulses).

For determination of *NR2F6* promoter activity, cells were co-transfected with 15 μg human *RARA*/*RXRA* expression constructs in pEF1a-Neo (or empty vector, with tags as indicated) together with human proximal *NR2F6* promoter-driven firefly luciferase reporters (15 μg), and pTK-Renilla-Luc (0.3 μg; Promega) for normalization.

For determination of TCF/LEF response element activity, cells were co-transfected with 15 μg of HA-tagged human *TCF7* (isoform 4, dominant-negative) or *NR2F6* expression constructs (wild-type or DNA-binding-deficient C111S mutant) cloned into pEFa-Neo, or the corresponding empty-vector control, together with 10 μg of TOP or FOP TCF/LEF response element-driven firefly luciferase reporter plasmid (Promega, 9PIE461). For normalization, 0.1 μg of pTK-Renilla-Luc (Promega) was included in all transfections. Cells were stimulated overnight with 50 ng/mL phorbol 12,13-dibutyrate and 500 ng/mL ionomycin (PdBu/iono; Sigma, P1269 and I0634). Where indicated, cells were treated with 7 μM of the GSK inhibitor SB415286 (Selleckchem, S2729), added directly to the culture medium.

Luciferase activities were quantified using the Dual-Luciferase Reporter Assay (Promega, E1960) on a PHERAstar FS plate reader (BMG LABTECH).

#### Design of stable CRISPR-Cas9-mediated NR2F6 KO

*NR2F6* was targeted using three pre-designed sgRNAs: sgRNA1 (target sequence: 5′-TACGGTGTCTTCACCTGCGA-3′, exon 1, forward strand), sgRNA2 (target sequence: 5′-GTACTGCGCCCGCACATACT-3′, exon 3, reverse strand), and sgRNA3 (target sequence: 5′-AGGTGTAGCTGAGGTTGCGG-3′, exon 1, reverse strand). Control sgRNAs included a non-targeting control (NTC; hypothetical target sequence: 5′-GCTCTCGTACGGCGCGTATC-3′) (all Dharmacon; SG-003423–01/02/03) or a safe-harbor (SH) sgRNA targeting *PPP1R12C* (target sequence: 5′-CGTTGCCAGTCTCGATCCGC-3′, intron 1), designed using Benchling. sgRNA specificity was verified using Cas-OFFinder.

#### *In vitro* cleavage assay

Genomic DNA (gDNA) was isolated from CD3^+^ T cells using the NucleoSpin Tissue Kit (Macherey-Nagel, 740952) according to the manufacturer’s instructions, and DNA concentrations were determined with a DeNovix DS-11 spectrophotometer. To generate DNA cleavage templates, 200 ng of gDNA were used to amplify *NR2F6* genomic regions targeted by the respective sgRNAs. PCR was performed using GoTaq G2 Hot Start Polymerase with the corresponding buffer, 2 mM MgCl_2_, (all from Promega, M7401, M891A and A351H) and 200 nM dNTPs (Biozym, 331520). The primer pairs used were 5′-ACGAACGGCGTGGACAAG-3′ and 5′-CCAAGTTCCACGTTCGATTCC-3′ for the region in exon 1 targeted by sgRNA1 and sgRNA3 (638 bp amplicon), and 5′-CGATCCTCCCATTAGCCTTC-3′ and 5′-TTCCCTGTCCCTTGAGGTCT-3′ for the region in exon 3 targeted by sgRNA2 (773 bp amplicon) (all from Eurofins Genomics). PCR cycling conditions consisted of an initial denaturation at 95°C for 2 min followed by 35 cycles of 95°C for 45 s, 62°C for 30 s, and 72°C for 60 s, and a final extension at 72°C for 5 min. PCR products were purified using the Monarch PCR & DNA Cleanup Kit (New England Biolabs, T1030L) and subjected to *in vitro* cleavage by incubating 100 ng of purified template DNA with 250 ng Cas9 endonuclease, 0.67 μM target-specific or SH sgRNA, 1× Cas9 reaction buffer, and 1× BSA (all from Takara Bio, 632639) in a 15 μL reaction at 37°C for 60 min. Reactions were terminated by heating to 80°C for 5 min, and cleaved DNA fragments were analyzed by agarose gel electrophoresis and visualized using peqGREEN DNA dye on a GelDoc Go imaging system (Bio-Rad).

#### Validation of *NR2F6*-targeting sgRNAs in HEK293T cells

HEK293T cells (Abcam, ab255449) were cultured in DMEM (PAN-Biotech, P04-01500) supplemented with 10% FBS (Biowest, S1810-500), 100 U/mL penicillin/100 μg/mL streptomycin (PAN Biotech, P06-07100), and 2 mM L-glutamine (PAN Biotech, P04-80050). Cas9-sgRNA ribonucleoprotein (RNP) complexes were assembled by incubating recombinant *S. pyogenes* Cas9 nuclease (IDT, 1081059, NLS- and 6× His-tagged) with sgRNAs targeting *NR2F6* or an SH control at a 1:2.5 M ratio (90 pmol Cas9, 225 pmol sgRNA) for 20 min at 25°C. RNPs were delivered by Amaxa nucleofection using the Cell Line Nucleofector™ Kit (Lonza, VCA-1003) and program Q-001. Knockout efficiency was assessed 24 h post-electroporation by immunoblot analysis of NR2F6 protein levels in whole-cell lysates.

#### Lentiviral *NR2F6* KO, *NR2F6* OE & CAR expression

*NR2F6* knockout (KO) was achieved using EF1α-driven Cas9-T2A-puromycin and U6-driven sgRNAs targeting *NR2F6* (sgRNA2 or sgRNA3) or a non-targeting control (all from Dharmacon, GSGH11935-247640844, GSGH11935-247620068 and GSGC11963). Stable *NR2F6* overexpression (OE) was generated using EF1α-driven *NR2F6* or control construct encoding β-galactosidase (aa 2–83) linked via an IRES to puromycin resistance (VectorBuilder, VB241126-1331hgg and VB241127-1225nsn). CAR expression employed a second-generation EGFR-specific construct comprising a Cetuximab-derived scFv, CD8 hinge and transmembrane domains, 4-1BB costimulatory domain, and CD3ζ signaling domain (Creative Biolabs, CAR-MZ007).

Third-generation self-inactivating lentivirus was produced in HEK293T cells as previously described by others.^38^ Briefly, 4–5 × 10^6^ HEK293T cells were seeded in DMEM (PAN Biotech, P04-03500) supplemented with 10% FBS (Biowest, S1810-500), 100 U/mL penicillin/100 μg/mL streptomycin (PAN Biotech, P06-07100), and 2 mM L-glutamine (PAN Biotech, P04-80050) in 100 mm tissue culture-treated dishes. At 70–90% confluency, cells were transfected in antibiotic-free medium using third-generation lentiviral packaging plasmids (Creative Biolabs, CART-027CL; 0.9 pmol pHELP1, 1.5 pmol pHELP2, 1.3 pmol pHELP3) together with 2.25 pmol transfer plasmid. Plasmids were diluted in 1.5 mL Opti-MEM (Thermo Fisher Scientific, 31985062). In parallel, Lipofectamine 2000 (Thermo Fisher Scientific, 11668019) was pre-diluted in Opti-MEM at a DNA (μg):Lipofectamine (μL) ratio of 1:1.3–1.6, then combined 1:1 with the DNA mix and incubated for 20–30 min at RT before dropwise addition (3 mL total) to cells. Medium was replaced after 6–18 h, and viral supernatants were collected up to 60 h post-transfection, cleared by centrifugation (500 rcf, 10 min, 4°C), and filtered through 0.45 μm cellulose acetate filters (Thermo Fisher Scientific, 10009230). Lentiviral particles were concentrated using Lenti-X™ Concentrator (Takara, 631232) at a 3:1 supernatant-to-reagent ratio (overnight for first harvest, ≥3 h for second harvest) followed by centrifugation (1500 rcf, 45 min, 4°C). Viral pellets were resuspended in RPMI to 1/100 of the original volume and used immediately or stored shock-frozen.

For stable genetic modification, CD3^+^ T cells were activated on day 0 using αCD3/αCD28 Dynabeads (Thermo Fisher Scientific, 11132D). Twenty-four hours later (day 1), cells were transduced in the presence of polybrene (8 μg/mL; Sigma, TR-1003-G). Transduced cells were selected with puromycin (2.5 μg/mL; Sigma, P8833) from days 3–5, followed by bead removal and expansion until day 16. Cultures were maintained continuously in cRPMI supplemented with 10 ng/mL IL-2 (BioLegend, 589102), refreshed every 2–3 days while adjusting cell density to 0.5–1 × 10^6^ cells/mL.

For simultaneous *NR2F6* modulation and CAR expression, cells were co-transduced with both vectors simultaneously. On day 14, CAR^+^ cells were enriched by recombinant EGFR-based MACS using MS columns and an OctoMACS Separator (Miltenyi, 130–042–201 and 130–042–109) based on manufacturer’s instruction protocols. All steps were performed at 4°C in CAR buffer (PBS, 2 mM EDTA, 2% BSA). Briefly, after a 15 min block, cells were labeled for 20 min with biotinylated EGFR (0.5 μg/mL; Creative Biolabs, CARD-LX025) and anti-biotin microbeads (2.5 μL per 10^6^ cells; Miltenyi, 130–090–485), magnetically separated, and the positive fraction collected. Sort purity was confirmed by flow cytometry. Cells were then expanded until days 22–23.

To generate NR2F6 knockout and overexpression Jurkat-TAg derivatives (Jurkat^NTC^, Jurkat^*NR2F6−*/–(sgRNA2)^, Jurkat^*NR2F6−*/–(sgRNA3)^, Jurkat^CTRL−OE^, and Jurkat^*NR2F6*−OE^), analogously to primary T cells, these were transduced in the presence of 8 μg/mL polybrene and maintained under continuous selection with 1 μg/mL puromycin.

#### CRISPRa-mediated endogenous NR2F6 induction

CRISPR activation (CRISPRa) was performed using a catalytically dead mutant of Cas9 nuclease (dCas9) fused to the VP64-p65-Rta (VPR) transcriptional activator complex together with gRNAs targeting the *NR2F6* promoter region or non-targeting control (NTC) sequences. An EF1α-driven dCas9-VPR construct linked via a P2A cleavage site to a puromycin resistance cassette (gift from Kristen Brennand, Addgene plasmid #99373) was stably introduced by lentiviral transduction followed by puromycin selection. Subsequently, cells were electroporated with 0.45 μM gRNAs using the Thermo Neon™ Transfection System (Thermo Fisher Scientific, N10096) according to the manufacturer’s instructions.

gRNAs were generated by annealing tracrRNA (Dharmacon, U-002005–50) with crRNAs at a 1:1 M ratio by denaturation at 95°C for 5 min followed by gradual cooling to room temperature. A pool of four crRNAs targeting the *NR2F6* promoter (Dharmacon, P-003423-01-0020; target sequences: 5′-GGCCGCCCTTATAAGGGCAT-3′, 5′-CAGGGGTTAAGGCCCGAGCT-3′, 5′-TTAAGGCCCGAGCTGGGCGA-3′, and 5′-GAGGAAGCAAACACAATCTA-3′) or four NTC crRNAs (Dharmacon, U-009500–10–20; hypothetical target sequences: 5′-GATACGTCGGTACCGGACCG-3′, 5′-GTAACGCGAACTACGCGGGT-3′, 5′-GTCGACGTTATTGCCGGTCG-3′, and 5′-GGAAACCTACGTCGACGAAT-3′) was used.

For primary human T cells, lentiviral transduction and puromycin selection were performed analogously to the CRISPR-mediated knockout or stable overexpression protocols described above. After expansion until day 17, cells were stimulated with plate-bound anti-CD3 antibodies (1 μg/mL) to enhance dCas9-VPR expression, followed by electroporation with gRNAs 24 h later.

For Jurkat^dCas9−VPR^ cells, puromycin selection was maintained continuously at 1 μg/mL following lentiviral transduction before electroporation with gRNAs.

#### Flow cytometry

Flow cytometry was performed on a FACSCanto II (4-2-2 configuration; BD Biosciences) as previously described.^39^ Briefly, cells were stained with a live/dead dye (BD Biosciences, 65–0865–14) diluted 1:2000 in HBSS for 10 min at RT, followed by surface staining for 20 min at 4°C with the following antibodies (all 1:50; BioLegend): CD3-AF488 (300415), CD4-Pacific Blue (317424), CD8-BV510 (301048), CD25-PerCP-Cy5.5 (356112), CD45RA-PE-Cy7 (304126), CD45RO-AF488 (304212), CD69-AF647 (310918), LAG-3-PE-Cy7 (369310), PD-1-PE (329906), and TIM-3-PerCP-Cy5.5 (345016).

For intracellular cytokine detection, brefeldin A and monensin (GolgiPlug/GolgiStop; 1:1000/1:1500; BD Biosciences, 555029 & 554724) were added 45 min post-stimulation; cells were then fixed for 30 min (BioLegend, 420801), and all subsequent steps were performed in permeabilization/wash buffer (BioLegend, 421002) with an antibody mix (surface + intracellular) incubated for 20 min, including granzyme B-FITC (515403), IFN-γ-PE-Cy7 (502528), and IL-2-PE (500307).

For CAR detection using biotinylated recombinant EGFR (rEGFR; Creative Biolabs, CARD-LX025), all steps were performed in CAR buffer (PBS, 2% BSA, 2 mM EDTA) with 1 μg/mL rEGFR included in the antibody mix, followed by staining with BV421-conjugated streptavidin (BioLegend, 405226) for 20 min.

Data were analyzed in FlowJo v10.7.2; unless otherwise stated, analyses were performed after pre-gating on viable cells, lymphocytes (FSC-A vs. SSC-A), and singlets (FSC-A vs. FSC-H or FSC-W).

#### (CAR-) T cell restimulation *in vitro*

T cells were stimulated polyclonally using plate-bound anti-CD3 antibodies (clone OKT3; Bio-X-Cell, BE0001-2; coated overnight in PBS) with soluble anti-CD28 antibodies (clone 9.3; Bio-X-Cell; BE0248) at indicated concentrations. CAR-mediated stimulation was performed in streptavidin-coated flat-bottom 96-well plates (50 μL of 10 μg/mL streptavidin in a.d.; Thermo Fisher Scientific, 21125; evaporated at RT) loaded with biotinylated recombinant EGFR (1 μg/mL; Creative Biolabs, CARD-LX025) for 20 min at 4°C before seeding CAR-T cells. Where indicated, Jurkat-TAg cells (or derivatives) were stimulated with 50 ng/mL phorbol 12,13-dibutyrate and 500 ng/mL ionomycin (PdBu/iono; Sigma, P1269 and I0634), added directly to the culture medium.

#### CAR-T cell killing assay

A549^*B2M−/−*^ ^eGFP^ or A549^*B2M−/−*^ ^mCherry^ target cells (5 × 10^5^) were seeded into 96-well plates 12–16 h before addition of effector cells at a 1:1 effector-to-target ratio (200 μL cRPMI). 100 μL of supernatant were replaced with fresh medium containing tumor cells after 48 h (all in triplicates). Target cell killing was monitored by live-cell imaging using an Incucyte SX5 system (Sartorius). Fluorescent images (GFP or mCherry) were acquired at 10× magnification (4 fields per well, every 2 h). Fluorescence was quantified using Incucyte 2022B software as total integrated intensity, using predefined segmentation parameters (Surface Fit with threshold of 1 calibrated unit; edge split off enabled; minimum area 5 μm^2^). Where indicated, supernatants were collected for Bio-Plex analysis and cells harvested for flow cytometry.

#### RT-qPCR & bulk RNA-sequencing

Total RNA was isolated using the RNeasy kit (Qiagen, 74016) according to manufacturer’s instructions. Complementary DNA (cDNA) to isolated RNA was transcribed using Omniscript RT Kit (Qiagen, 205111). For this, isolated RNA was mixed with the following reagents to achieve 40 μL of a final reaction set-up with 1× RT buffer, 0.2 μM of each dNTP, 0.1 U/μL reverse transcriptase (all from kit), 20 pg/μL oligo-dT 15 primer (Promega, C1101), 20 pg/μL random hexamer primer (Promega, C1181) and 4 U/μL ribonuclease inhibitor (Promega, N2511). The reaction was incubated at 37°C for 1 h before heat-inactivation for 6 min at 95°C.

TaqMan-based reverse transcriptase - quantitative PCR (RT-qPCR) was performed as described previously^39^ using LUNA Universal Probe qPCR Master Mix (NEB, 3004) together with following primers: *RPL13A* (Hs04194366_g1_VIC_MGB_PL) and *NR2F6* (Hs00172870_m1_FAM_MGB) (both from Thermo Fisher Scientific/Applied Biosciences). The reaction was run in a 7500 Fast Real-Time PCR instrument (Applied Biosystems) with an initial denaturation (95°C, 60 s) followed by 40 cycles of denaturation (95°C, 15 s) and extension (60°C, 30 s).

Bulk RNA-Seq libraries were prepared using a non-strand-specific poly(A)-enriched protocol (Novogene, PT042). Briefly, mRNA was selectively captured from total RNA using poly-T oligo-attached magnetic beads. After fragmentation, cDNA synthesis was carried out in two steps: first-strand synthesis with random hexamer primers, followed by second-strand synthesis. The library was then constructed through a series of steps, including end repair, A-tailing, adapter ligation, size selection, amplification, and purification. To ensure quality, Qubit and real-time PCR were used for quantification, while a bioanalyzer assessed size distribution. Libraries were subjected to sequencing on an Illumina NovaSeq X Plus platform (paired-end, 150 bp; ≥9 GB per sample) at Novogene.

Reads were quality-filtered with *fastp* and aligned to the human reference genome (GRCh38/hg38) using *HISAT2* (v2.2.1). Raw counts were restricted to protein-coding genes and prefiltered to retain features with ≥10 total counts across all samples. Differential expression analysis was performed using *DESeq2* (v1.42.1),^37^ with genotype as the primary variable and donor effects (and CD4:CD8 ratio for *NR2F6* overexpression) included as covariates. Rlog-transformed data were used for visualization in heatmaps (showing Z-scored expression values – per donor in case of *NR2F6* knockout). GSEA was performed using *fgsea* (v1.28.0) on genes ranked by log2 fold change, using selected KEGG pathways or a custom TCF-1 targets gene set. Transcription factor activity was inferred with *decoupleR* (v2.8.0)^36^ using the CollecTRI human TF network, considering only TF–target sets with ≥5 genes and deriving genotype contrasts from the *DESeq2* Wald test statistic. Data visualization was performed using *ggplot2* (v3.5.1) and *pheatmap* (v1.0.12).

#### ATAC-seq processing, differential accessibility, and motif enrichment analysis

Assay for Transposase-Accessible Chromatinusing Sequencing (ATAC-Seq) libraries were prepared and sequenced by GENEWIZ (AZENTA Life Sciences) on an Illumina platform (2 × 150 bp). Adapter trimming and quality filtering were performed using *Trimmomatic* (v0.38). Reads were aligned to the human reference genome (hg38) using *Bowtie2*. Alignments were filtered with *SAMtools* (v1.9) to retain primary, concordantly mapped reads with MAPQ ≥30. PCR and optical duplicates were removed using *Picard* (v2.18.26). Mitochondrial reads and reads mapping to unplaced contigs were excluded prior to downstream analyses.

Accessible chromatin regions were identified using *MACS2* (v2.1.2), excluding blacklisted genomic regions where available. Peaks were merged across biological replicates within each condition, retaining those present in at least 66% of samples per group. Condition-specific peak sets were then merged to generate a consensus peak set, and read counts were quantified across all samples. Differential chromatin accessibility between *NR2F6* OE and CTRL conditions was assessed using *DiffBind*, with significantly differential peaks defined as FDR ≤0.05.

Transcription factor (TF) motif enrichment analysis was performed on significant differential peaks (FDR <0.05) using *monaLisa* (v.1.16.0).^27^ Peaks were restricted to standard chromosomes and resized to 200 bp centered on the peak midpoint. Genomic sequences (hg38) were extracted using BSgenome.Hsapiens.UCSC.hg38. TF motifs were obtained from JASPAR2022 and converted to position weight matrices. Peaks were classified according to differential accessibility between *NR2F6* OE and CTRL based on log2 fold change (CTRL-enriched: log2FC < −0.5; *NR2F6* OE-enriched: log2FC > 0.5). Motif enrichment was computed separately in each class. Differential motif enrichment was defined as the difference in monaLisa log2 enrichment scores between *NR2F6* OE- and CTRL-enriched peaks (OE − CTRL), where positive values indicate preferential motif enrichment in *NR2F6* OE-associated accessible regions and negative values indicate enrichment in CTRL-associated regions. Selected transcription factor families, including NFAT, NF-κB, and AP-1, were visualized in a volcano plot showing motif enrichment contrast together with the corresponding adjusted *p* values for enrichment in CTRL or *NR2F6* OE, respectively.

#### Immunoblot & EMSA

Whole-cell lysates (WCL) were prepared in RIPA buffer (Sigma, R02789) supplemented with 1× Halt protease/phosphatase inhibitor and 5 mM EDTA (Thermo Fisher Scientific, 78444), lysed on ice for 30 min with intermittent vortexing, then cleared by centrifugation (21,000 rcf, 15 min, 4°C) to collect the protein-containing supernatant. Nuclear extracts (NE) were generated using a standard two-buffer method: cells were pelleted (9,500 rcf, 1 min), washed with ice-cold PBS, and resuspended in 300 μL buffer 1 (10 mM HEPES pH 7.9, 10 mM KCl, 0.1 mM EDTA, 0.1 mM EGTA, 1 mM DTT, 0.5 mM PMSF) for 15 min on ice; 20 μL 10% NP-40 was then added, samples were vortexed and spun (2,350 rcf, 5 min) to disrupt the plasma but not nuclear membrane. The supernatant (cytoplasmic fraction) was frozen, and the nuclear pellet was washed twice with buffer 1 (2,350 rcf, 5 min), resuspended in buffer 2 (20 mM HEPES pH 7.9, 0.4 M NaCl, 1 mM EDTA, 1 mM EGTA, 1 mM DTT, 1 mM PMSF), and incubated for 30 min with shaking. After centrifugation (13,500 rcf, 10 min), the supernatant containing nuclear proteins was collected. Protein concentrations were determined by BCA assay (Thermo Fisher Scientific, 23227) using a PHERAstar FS plate reader (BMG LABTECH).

Immunoblotting was performed either using a Jess Simple Western system (ProteinSimple) according to the manufacturer’s instructions or by conventional SDS-PAGE and immunoblotting as described previously.^40^ Briefly, lysates were mixed with SDS loading buffer, heated (95°C, 5 min), separated on 4%–12% Bis-Tris gels in MOPS buffer, and transferred to methanol-activated PVDF by semi-dry transfer. Membranes were blocked (5% milk in TBST, 1 h), incubated overnight at 4°C with primary antibodies, then washed and incubated for 1 h at room temperature with HRP-conjugated secondary antibodies. The following primary antibodies were used: anti-β-actin (Santa Cruz, sc-1615; 1:3000), anti-HDAC1 (Santa Cruz, sc-81598; 1:1000), anti-NR2F6 (ProteinTech, 60117-2-Ig; 1:500), anti-NFATc1 (Santa Cruz, sc-7294; 1:500), anti-phospho-tyrosine (Cell Signaling, 96215; 1:500), and anti-TCF-1 (Santa Cruz, sc-101170; 1:1000).

Electrophoretic mobility shift assays (EMSAs) were performed as previously described^41^ using consensus oligonucleotides for AP-1 (5′-CGCTTGATGACTCAGCCGGAA-3′), NF-κB (5′-CTGGGGACTTTCCGCT-3′), and TCF/LEF (TOP: 5′-TGTTAAGATCAAAGGGTTTAAGATC-3′; FOP: 5′-TGTTAAGAAAAAAGGGTTTAAGATC-3′; corresponding to the TOP/FOPFlash reporter sequences). All oligonucleotides were custom-synthesized by custom-synthesized by Eurofins MWG Operon. Briefly, probes were 32P end-labeled with γ-[32P]ATP using T4 polynucleotide kinase, purified on G-25 columns, and incubated with nuclear extracts in binding buffer (40 mM Tris, 200 mM NaCl, 4 mM EDTA, 20% glycerol, 4 mM DTT, 0.4% BSA, 170 μg/mL poly(I:C) in a.d.) plus Stabilizing Solution D (Activ Motif, 37488) at 4°C for 20 min. DNA–protein complexes were resolved on 5% native polyacrylamide gels in 0.5× TBE at 300 V, gels were dried, and signals were detected by autoradiography.

Brightness and contrast of gel images were adjusted to improve band visibility. Any adjustments were applied consistently across all conditions being compared (e.g., within each donor). The corresponding contiguous image regions are indicated by black borders in the figure.

#### Bio-plex technology

Concentration of IL-2, IFN-γ and GZMB from cell culture supernatants were determined using Luminex xMAP technology on a Bio-Plex 200 device (BioRad) by combination of corresponding kits (Biorad; 171-B5003M and 171-B5019M and ThermoFisher, EPX01A-12027–901 for IL-2, IFN-γ or GZMB, respectively) based on manufacturer’s instructions.

### Quantification and statistical analysis

Statistical analyses were performed in GraphPad Prism (v10.2.3) or R (v4.3.3.) and are specified together with the sample size in the respective figure legends. Normally distributed data were analyzed using paired or unpaired *t*-tests as appropriate, or one-way or two-way ANOVA (or mixed-effects models when missing values were present) followed by Holm–Šidák post hoc correction; *p* < 0.05 was considered significant, and data are presented as mean ± SEM (for independent biological replicates i.e., donors individual points are additionally displayed and connected by lines) unless stated otherwise. Asterisks used in the figures indicate statistical significance as follows: n.s. = not significant, ∗*p* < 0.05, ∗∗*p* < 0.01, ∗∗∗*p* < 0.001, ∗∗∗∗*p* < 0.0001. Of note, no correction for multiple testing was applied when comparing control samples to *NR2F6* knockouts generated using either sgRNA2 or sgRNA3, as the consistent effects observed with both sgRNAs were considered biologically robust and sufficient to mitigate the risk of false positives. Additionally, mathematical corrections for multiple testing were made only within individual markers across groups, rather than across multiple markers within one genotype, reflecting the primary focus on within-marker group differences.
