# Supplementary material for: NR2F6 functions as a key transcriptional checkpoint enforcing dysfunction of human CD3+ T cells and limiting CAR-T cell potency
Source: iScience. 2026 Oct 26;29(11):117982. doi: 10.1016/j.isci.2026.117982 (PMC13734946; doi:10.1016/j.isci.2026.117982)
Supplement: Supplementary file 1 [file mmc1.pdf]

iScience, Volume *29*

Supplemental information

NR2F6 functions as a key transcriptional checkpoint enforcing dysfunction of human CD3^{+} T cells and limiting CAR-T cell potency

Jiří Koutník, Thomas Gruber, Dorothee Freimark, Dominik Humer, Daniel Schreiber, Isabel Hölzl, Viktor Lang, Nino Tokic, Tajana Sajinovic, Dominik Wolf, Nikolaus Thuille, Kerstin Siegmund, Victoria Klepsch, and Gottfried Baier

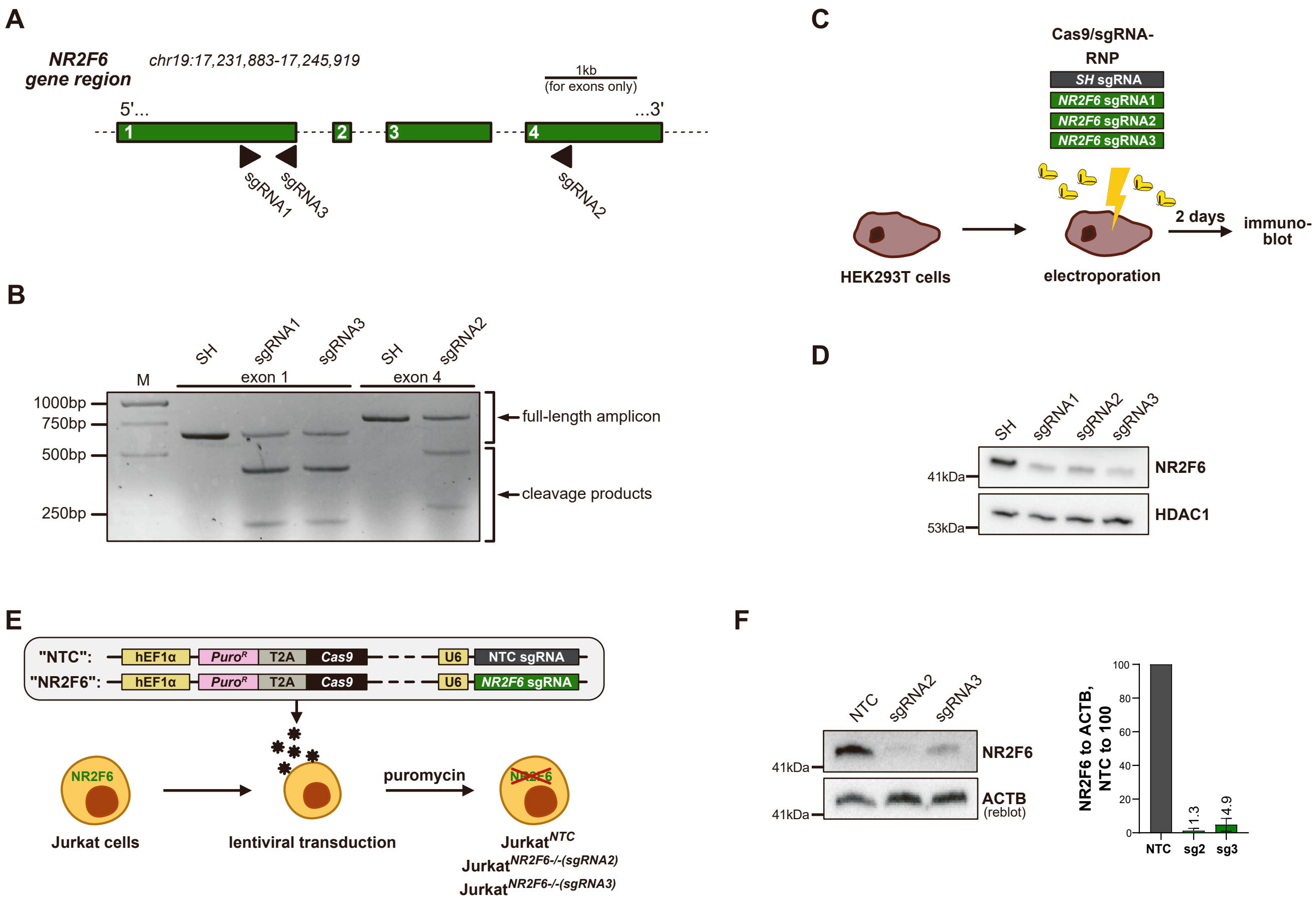

Figure S1. Establishment of efficient genetic deletion of human *NR2F6*

(A) Illustration of the genomic *NR2F6* region together with selected targeting sgRNAs.

(B) *In vitro* cleavage assay showing Cas9-mediated cleavage of PCR amplicons spanning the sgRNA target region. SH = safe harbor control, M = marker. (n = 1).

(C–D) Schematic overview of proof-of-concept experimental setup. HEK293T cells were electroporated with Cas9/sgRNA-RNPs followed by **(D)** analysis of *NR2F6* expression on protein level. (n = 1).

(E–F) Validation of complete *NR2F6* knockout by stable integration of Cas9 with corresponding sgRNA into Jurkat cells followed by antibiotic selection.

(E) Schematic overview of the workflow and lentiviral construct used.

(F) Western blot analysis of *NR2F6* knockout. A representative blot (left) and quantification (right) are shown. (n = 5 replicates from 4 passages).

All data are represented as mean ± SEM, with mean values indicated above the bars.

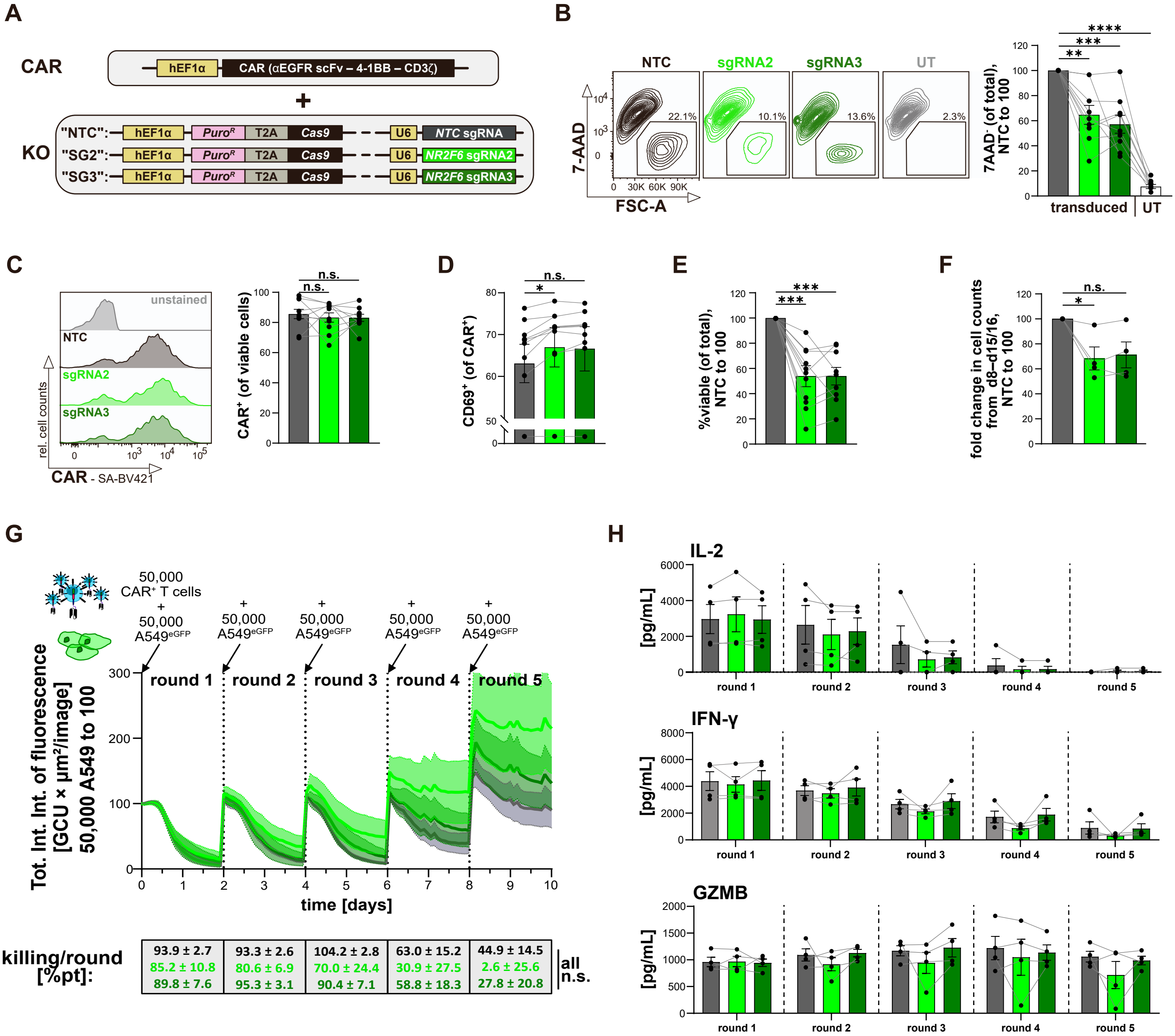

Figure S2. Loss of *NR2F6* abrogates hyperactivation-driven benefits in CAR-T cells by compromising survival

(A) Schematic overview of constructs of used CAR as well as *NR2F6* knockout or corresponding control.

(B) Indirect confirmation of successful puromycin selection on day 8 via 7-AAD viability staining analysed by flow cytometry. Representative contour plots and quantification are shown. UT = untransduced control. (n = 9 donors, paired t-test).

(C–E) Flow cytometric confirmation of **(C)** CAR expression, **(D)** the activation marker CD69 and **(E)** the cells' viability at the end of CAR-T cell production. (n = 10 donors, paired t-test).

(F) Fold change in cell numbers after puromycin selection for 1 week (n = 4–5, paired t-test).

(G) Serial killing assay: CAR-T cells were co-cultured with $A549^{B2M-/-\ eGFP/mCherry}$ target cells at an effector-to-target (E:T) ratio of 1:1. Every 48 h, fresh $A549^{B2M-/-\ eGFP/mCherry}$ cells were added at the same number as initially seeded. Tumour cell killing was monitored by live-cell imaging every 2 h. (n = 5–6 donors, mixed effect analysis followed by Holm-Šídák correction for multiple testing).

(H) Bioplex analysis measuring IL-2, IFN-γ and GZMB concentrations from supernatant of co-culture at the end of every round of killing, prior to re-stimulation with fresh tumour cells. (n = 4 donors. repeated measure two-way ANOVA followed by Holm-Šídák correction for multiple testing).

n.s. = not significant. *p < 0.05, **p < 0.01, ***p < 0.001, ****p < 0.0001. All data are represented as mean ± SEM together with individual data points.

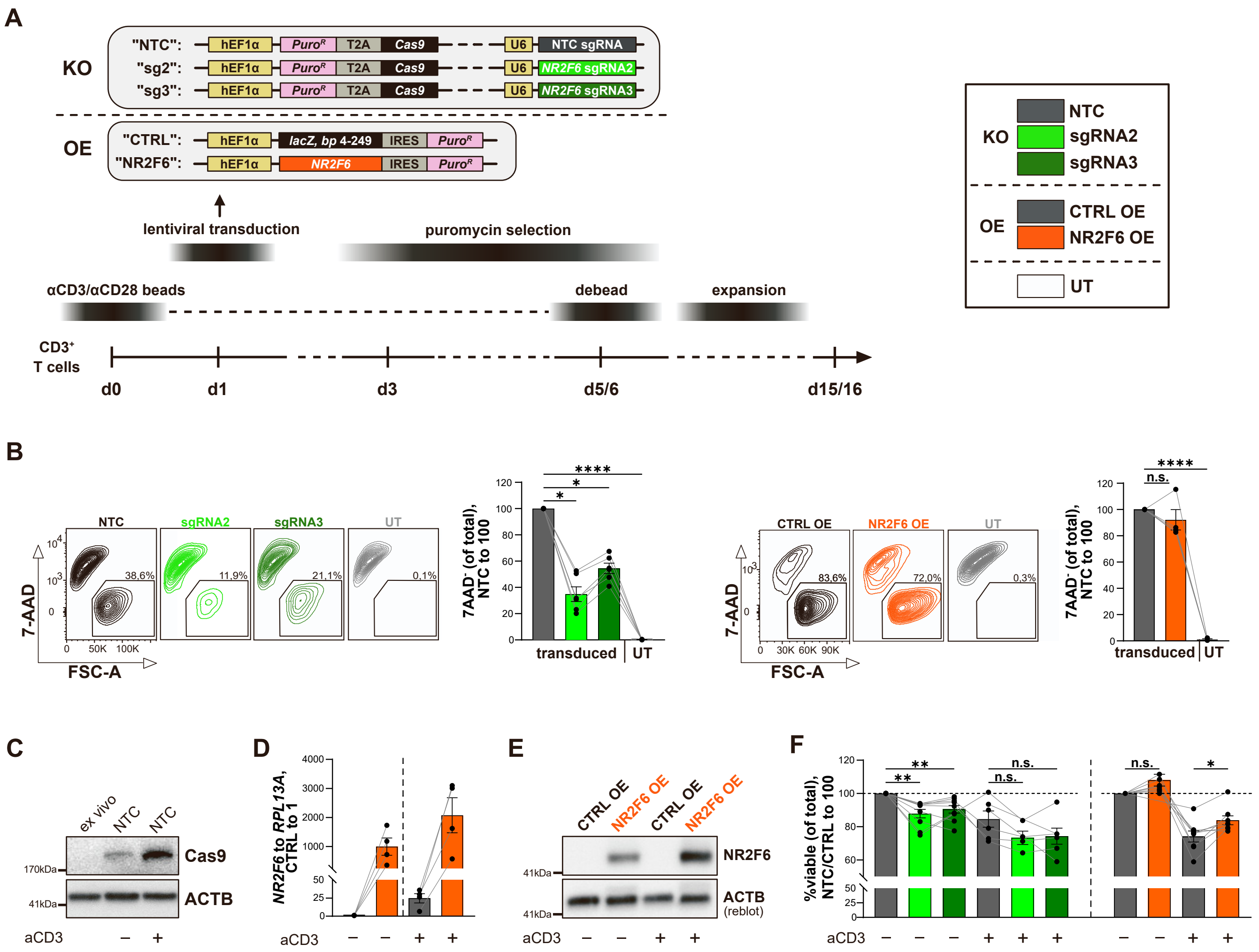

Figure S3. Generation and confirmation of stable *NR2F6* KO or OE in primary human T cells

(A) Schematic of the experimental workflow. CD3+ T cells were pre-stimulated via anti-CD3/anti-CD28 beads, transduced with the respective lentivirus as shown in the figure (mediating either KO or OE), selected via puromycin, debeaded and expanded up to day 16.

(B) Indirect confirmation of successful puromycin selection on day 8 via 7-AAD viability staining analysed by flow cytometry. Representative contour plots and quantification are shown. UT = untransduced control. (n = 4–6 donors).

(C) Representative immunoblot confirming Cas9 integration in *NR2F6* KO cells.

(D+E) Direct confirmation of *NR2F6* overexpression (at the end of production and upon 24 h re-stimulation with anti-CD3 antibodies — 0.3125 µg/mL or 5 µg/mL for KO or OE cells, respectively) via **(D)** RT-qPCR and **(E)** representative immunoblot. (n = 4 donors).

(F) Flow cytometric analysis of viability (at the end of production and upon 24 h re-stimulation with anti-CD3 antibodies — 0.3125 µg/mL or 5 µg/mL for KO or OE cells, respectively). (n = 4–15 donors).

n.s. = not significant. *p < 0.05, **p < 0.01, ***p < 0.001, ****p < 0.0001, from paired two-tailed t-test. All data are represented as mean ± SEM together with individual data points.

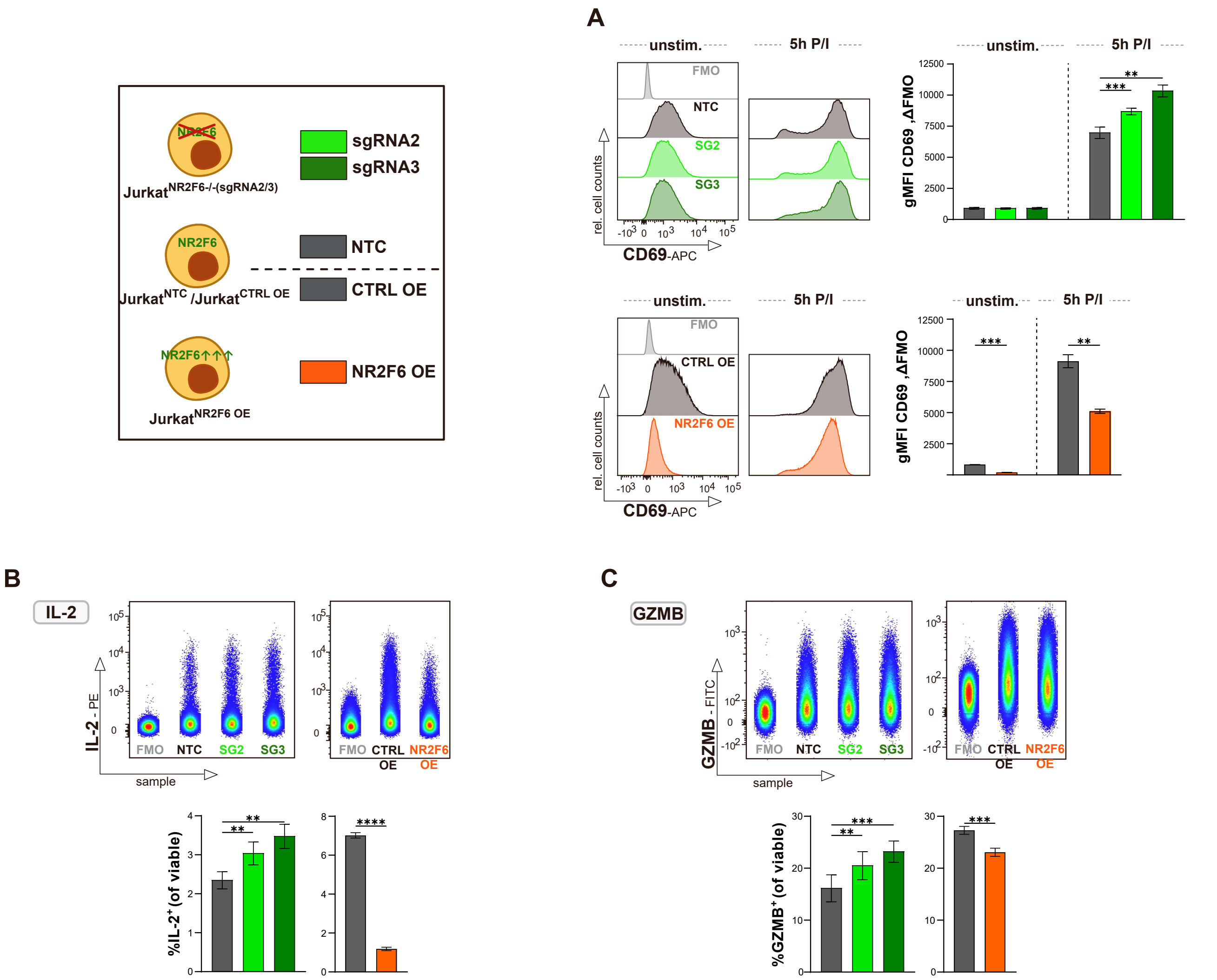

Figure S4. NR2F6 regulates activation and cytokine production in Jurkat cells

Jurkat cells lacking NR2F6 (Jurkat$^{NR2F6\text{-}/\text{-}(sgRNA2)}$ (light green), Jurkat$^{NR2F6\text{-}/\text{-}(sgRNA3)}$ (dark green)) or overexpressing NR2F6 (Jurkat$^{NR2F6\text{-}OE}$ (orange)) along with their respective controls (Jurkat^{NTC} and Jurkat$^{CTRL\text{-}OE}$ (grey)) were stimulated via PDBu/ionomycin (P/I) for 5 h and investigated via flow cytometry.

(A) Surface expression of CD69 pre- and post-stimulation.

(B+C) Intracellular levels of **(B)** IL-2 and **(C)** GZMB after trapping via brefeldin A and monensin 1 h post stimulation. Data are represented as mean ± SEM. Representative flow cytometry plots/histograms and quantification are shown. (n = 11–15 replicates from 4–6 passages or n = 3–4 replicates from 1 passage for KO or OE cells, respectively. Paired t-tests, with replicate pairs randomly assigned within the same passage). n.s. = not significant. *p < 0.05, **p < 0.01, ***p < 0.001, ****p < 0.0001.

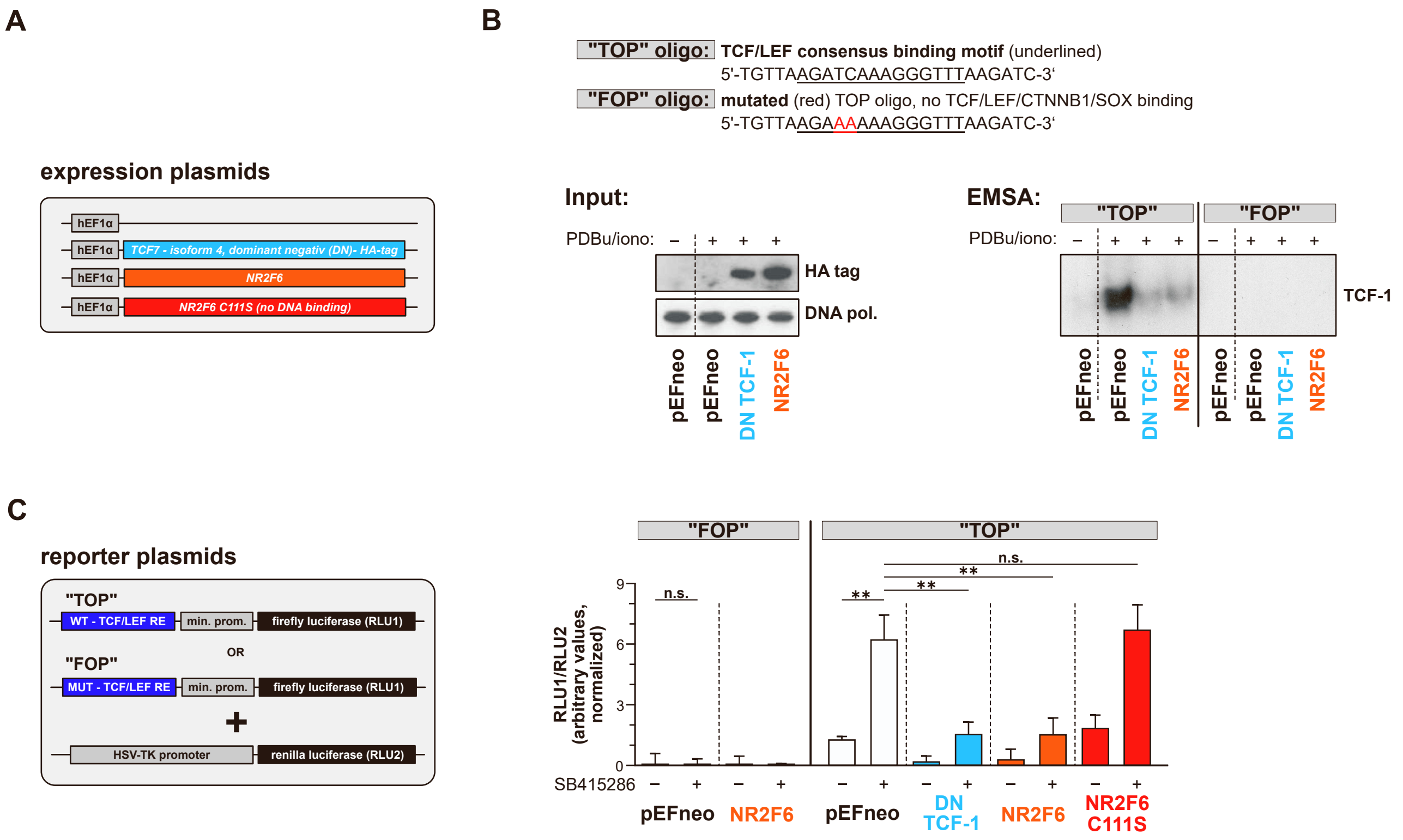

Figure S5. NR2F6 functionally interacts with TCF/LEF binding activity

Jurkat-TAg cells were electroporated with expression plasmids encoding wild-type ("wt") NR2F6, the DNA-binding mutant NR2F6 C111S, or dominant-negative TCF-1 (DN–TCF-1) prior to analysis of TCF/LEF function.

(A) Schematic overview of used expression plasmids.

(B) Representative EMSA showing TCF/LEF DNA-binding activity using either "TOP" or mutated "FOP" oligonucleotide probes following o/n stimulation with PDBu/ionomycin in the presence or absence of 7 µM GSK3 inhibitor (SB415286).

(C) TCF/LEF response element (RE) luciferase reporter assay. Expression plasmids shown in (A) were co-transfected with either TOP-RE or mutated FOP-RE minimal promoter-driven firefly luciferase reporter constructs (RLU1) together with the control reporter vector pTK-Renilla-Luc (RLU2). Cells were stimulated with PDBu/ionomycin and, where indicated, treated with 7 µM SB415286. Constructs (left) and quantification (right) of the RLU1/RLU2 ratio are shown.

Data represent mean ± SEM from n = 10 replicates. n.s. = not significant. *p < 0.05, **p < 0.01; unpaired t-test.

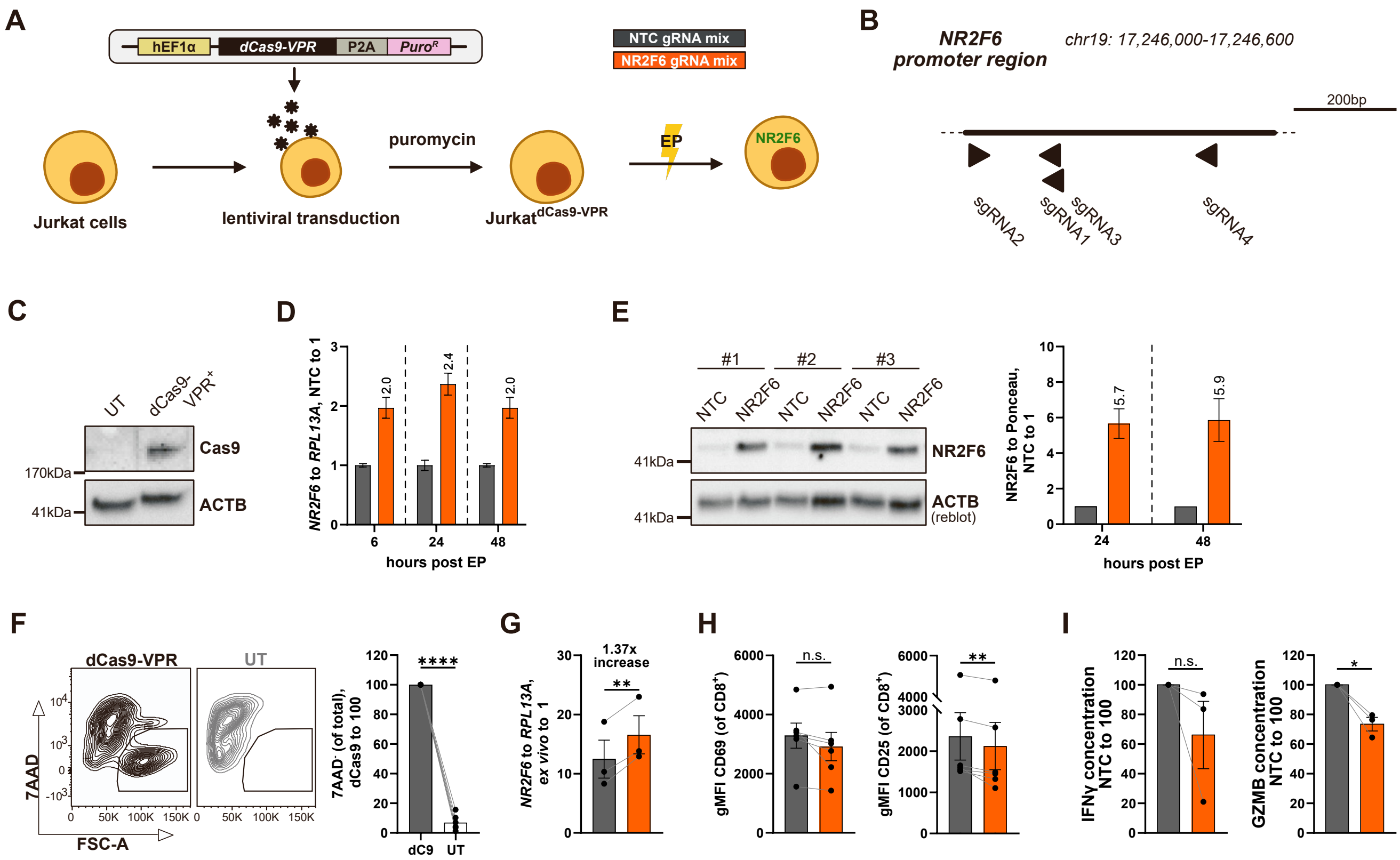

Figure S6. Modest genetic induction of *NR2F6* by CRISPRa weakens T cell activation and effector cytokine production

NR2F6 was upregulated using CRISPRa technology in either Jurkat cells **(A–E)** or human T cells **(F–I)** followed by analysis of activation and effector functions. Controls are represented in grey, *NR2F6*-upregulated cells are represented in orange.

(A) Schematic overview of the experimental set-up. Jurkat cells were lentivirally transduced to stably express dCas9-VPR, followed by puromycin selection and electroporation with a pool of gRNAs targeting the *NR2F6* promoter region.

(B) Illustration showing the genomic binding sites targeted by the selected gRNAs within the *NR2F6* promoter region.

(C) Western blot confirming expression of the dCas9-VPR fusion protein.

(D+E) Analysis of *NR2F6* expression following CRISPRa-mediated activation. **(D)** RT-qPCR and **(E)** western blot with representative blot (left) and quantification (right). n = 3–5 replicates from two independent electroporations. Data are represented as mean ± SEM, with mean values indicated above the bars.

(F–I) $CD3^+$ T cells were pre-stimulated via αCD3/αCD28 beads, transduced with a lentiviral vector encoding dCas9-VPR, selected via puromycin and debeaded. Following expansion, cells were re-stimulated via 1 µg/mL plate-bound anti-CD3 antibodies to boost transgene expression before electroporation with gRNAs targeting the *NR2F6* promoter region (orange) or a non-targeting control (NTC, grey) to induce its expression.

(F) Confirmation of successful puromycin selection on day 8 via 7-AAD viability staining analysed by flow cytometry. Representative contour plots and quantification are shown. UT = untransduced control.

(G) *NR2F6* expression 24 h post electroporation assessed by RT-qPCR.

(H) Flow cytometric analysis of activation markers CD69 and CD25 gated on $CD8^+$ T cells on day 19 upon 4 h re-stimulation via 5 µg/mL plate-bound anti-CD3 antibodies.

(I) Bioplex analysis determining the concentration of IFN-γ and GZMB in the culture medium 24 h post electroporation with gRNAs.

All data are represented as mean ± SEM together with individual data points. n = 3–6 donors with up to 3 replicates for re-stimulation per donor, from two independent experiments. n.s. = not significant. $^*p < 0.05$, $^{**}p < 0.01$, $^{***}p < 0.001$, $^{****}p < 0.0001$, from paired two-tailed t-test.

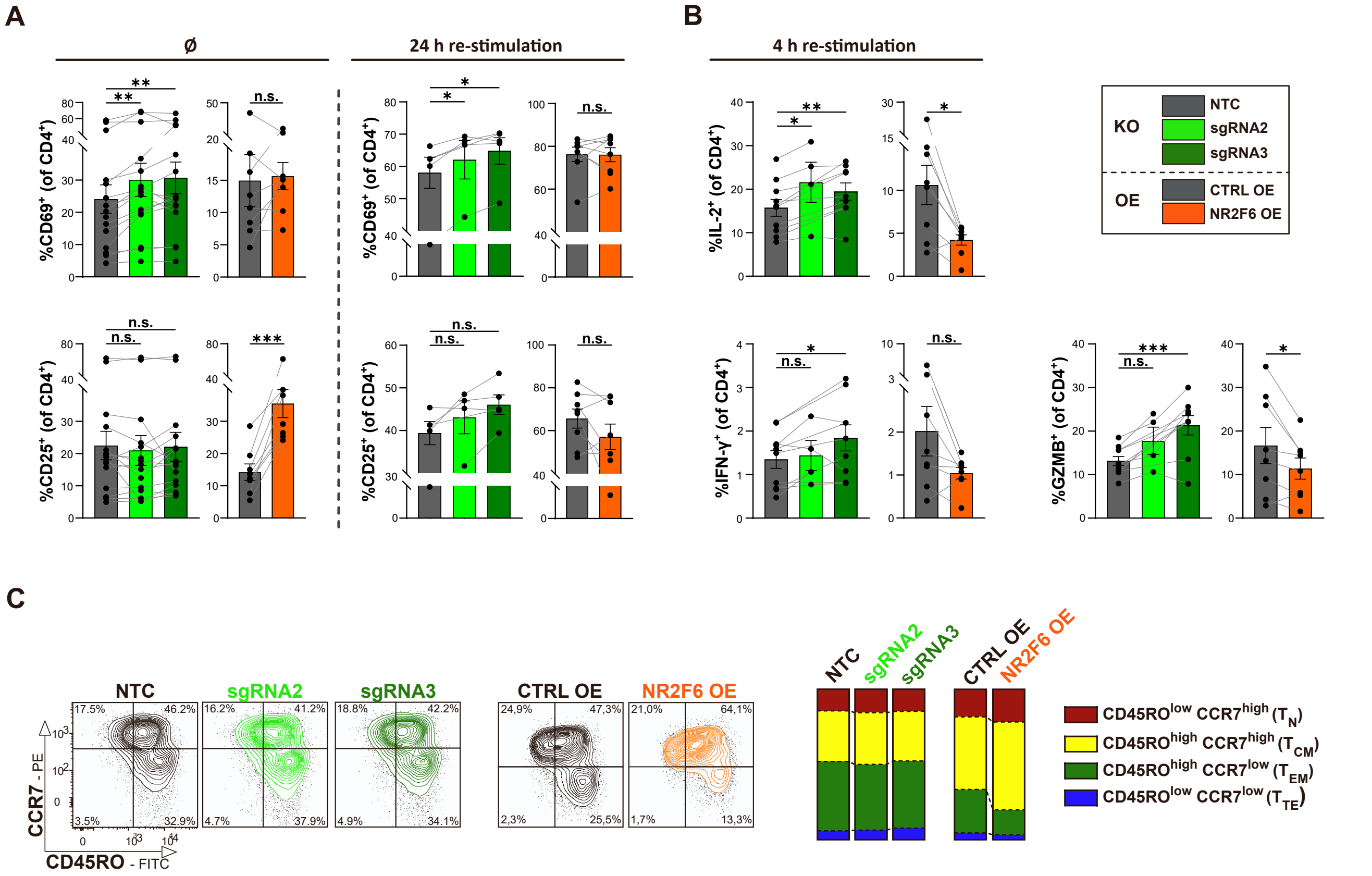

Figure S7. NR2F6's negative regulatory role on cytokine production and differentiation also applies to CD4⁺ T cells

Stably *NR2F6*-modified human T cells were analysed upon selection and 2-week expansion period. *NR2F6*-deficient cells are represented in green, *NR2F6* OE ones in orange.

(A) Flow cytometric characterization of the activation markers CD69 and CD25 pre-gated on CD4⁺ cells in resting cells and upon 24h re-stimulation via 0.3125 or 5 µg/mL (for KO or OE cells, respectively) plate-bound anti-CD3 antibodies. (n = 4–15 donors, mean ± SEM + individual data points, paired t-test).

(B) Intracellular flow cytometry analysis of of IL-2, IFN-γ and GZMB in CD4⁺ T cells upon 4h re-stimulation via 5 µg/mL anti-CD3 antibodies in the presence of brefeldin A and monensin (added 1 h post re-stimulation). (n = 4–8 donors, paired t-test). Data are represented as mean ± SEM together with individual data points.

(C) Flow cytometric characterization of the differentiation markers CCR7 and CD45RO (pre-gated on CD4⁺ T cells) and corresponding classification into naïve (T_N), central memory (T_{CM}), effector/memory (T_{EM}) and terminal effector (T_{TE}) T cells. Representative contour plots as well as quantification are shown. (n = 8–12 donors). *p < 0.05, **p < 0.01, ***p < 0.001.
